# In Vitro Effect of Ferruginol, Tanshinone, and Carnosol Analogues on the Proliferation of Three Breast Cancer Cell Lines

**DOI:** 10.3390/molecules30122529

**Published:** 2025-06-10

**Authors:** Miguel A. González-Cardenete, William E. Mendoza-Hernández, Sydney L. Lawson, Fatima Rivas

**Affiliations:** 1 Instituto de Tecnología Química, Universitat Politècnica de València-Consejo Superior de Investigaciones Científicas, Avda. de los Naranjos s/n, 46022 Valencia, Spain; wemenher@itq.upv.es; 2 Department of Chemistry, Louisiana State University, 133 Choppin Hall, Baton Rouge, LA 70803, USA

**Keywords:** abietane, cytotoxicity, breast cancer cells, tanshinone, carnosol, ferruginol

## Abstract

Ferruginol, tanshinones and carnosol are considered privileged natural products due to their demonstrated diverse biological activities with relevance to cancer research. Globally, cancer continues to be a major contributor to mortality rates, making these compounds potentially valuable molecular scaffolds for further development as potential anticancer agents. In this work, a focused library of ferruginol, tanshinone IIA, and carnosol analogues was studied to examine their effectiveness against various solid tumor models. The compounds were efficiently synthesized from either methyl 12-hydroxy-dehydroabietate or 12-hydroxydehydroabietylamine in 1–3 step processes with good chemical yields. The compounds that were synthesized underwent a methodical evaluation using multiple biological tests (including viability assays, clonogenic assays, and mitochondrial membrane polarization measurements) to determine their ability to inhibit in vitro the growth of three breast cancer cell linages. It was determined that while most compounds exhibited biological activity, compounds **10** and **11** demonstrated significant efficacy against triple negative breast cancer cells. These compounds continue to show promising biological activity, suggesting that additional studies to understand their mechanisms of action would be valuable.

## 1. Introduction

Cancer is one of the main public health problems around the world, responsible for almost one in sixth deaths worldwide [1]. This disease is the cause of around 30% of premature deaths in population aged 30–69 years which is specifically disproportional in women mainly because of female breast and cervical cancer [2]. According to the latest global cancer statistics, by world region for the year 2022, from the International Agency for Research on Cancer (IARC), there were nearly 20 million new cases alongside 9.7 million deaths from cancer in the year 2022, with breast cancer and lung cancer being the most frequent cancers in women and men, respectively [1]. Based on demographics, predictions indicate the number of new cases will rise to 35 million by 2050 [1].

Triple-negative breast cancer (TNBC) is a specific type of breast cancer that is characterized by the lack of three key receptors: estrogen receptors (ER), progesterone receptors (PR), and excess of HER2 protein. TNBC represents about 15–20% of the 2.3 million annual breast cancer diagnoses globally [1,3]. It tends to be more aggressive than other breast cancer types, and it is more commonly diagnosed in women under 40 years of age, and at a more advanced stage. TNBC has a less favorable prognosis than other breast cancer types and a higher risk of recurrence within the first 3–5 years after treatment [3]. It is, therefore, of great importance to invest in prevention and cancer treatments to save many lives worldwide. Chemotherapy is one of the conventional clinical therapies which many times shows limited success due to resistance and toxicity, leading to a continuous search for novel anticancer drugs. Plant-derived natural products have long been used as anticancer drugs representing almost 60% of FDA-approved antitumor drugs [4].

**Figure 1:**
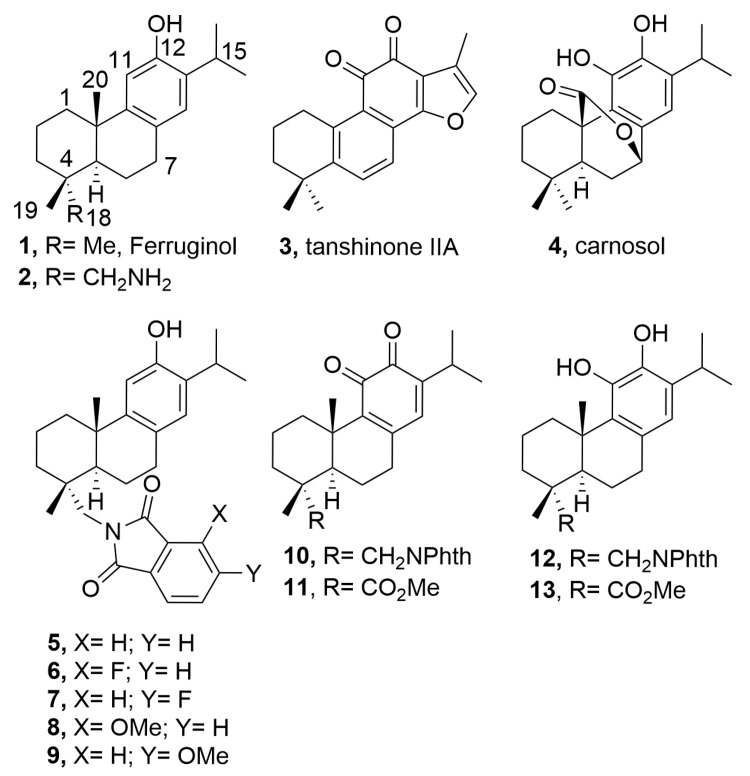
Chemical structure of abietanes **1**–**15**. Compounds **1**, **2** and **5**–**13** were submitted to MDA-MB-231, MCF07, and SUM149 cell growth inhibition tests.

Abietane-type diterpenoids are a large family of natural products found in hundreds of plants and characterized by a 20-carbon skeleton and a variety of oxygenation patterns which possess different pharmacological activities including anticancer properties [5,6]. The first isolated member, abietic acid, has been extensively studied, revealing several mechanisms involved in the antitumor properties [7]. Other members like ferruginol (**1**) [8] and its semisynthetic aminated analogue, 18-aminoferruginol (**2**) [9], along with tanshinone IIA (**3**) [10] and carnosol (**4**) [11] (Figure 1), have also demonstrated important antiproliferative and anticancer activities. The abietane natural products and their derivatives and analogues, thus, represent promising molecular prototypes for developing antitumor drugs.

**Scheme 1:**
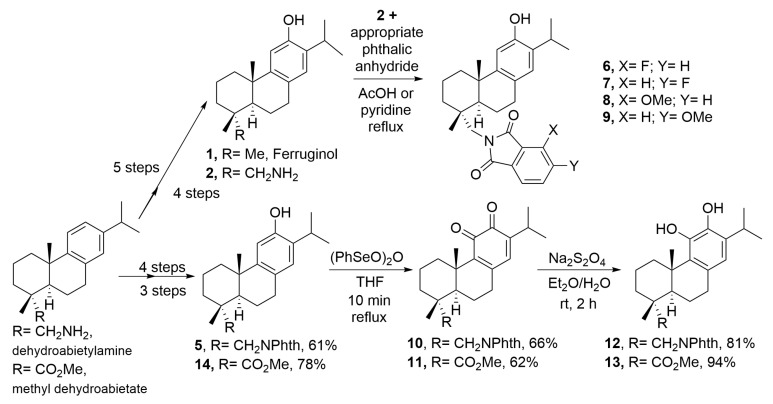
Synthetic sequence for the preparation of tested compounds **1**, **2** and **5**–**15** [14,15,16,17].

The rapid, uncontrolled growth and division of neoplastic cells is a fundamental hallmark of cancer diseases. Understanding cancer proliferation mechanisms continues to drive the development of targeted therapies aimed at disrupting specific aspects of this fundamental cancer process [12]. Encouraged by promising antiproliferative results reported by us on derivatizing readily available methyl 12-hydroxyabieta-8,11,13-trien-18-oate (**14**) (Scheme 1) and the phthalimide-ferruginol analogue **5** [9,13], we have synthesized further new and related known analogues. In this work, those new molecules, along with some studied previously, were assessed across a diverse panel of breast cancer cells and several biological tests (including viability assays, clonogenic assays, and mitochondrial membrane polarization measurements) to assess their ability to control growth in cancer cells and unveil their role in antiproliferative action.

## 2. Results

### 2.1. Chemistry

Compounds **1** (ferruginol) and **2** (18-aminoferruginol) (Figure 1) were synthesized following previously reported methods starting from the commercially available (+)-dehydroabietylamine [14,15,16]. The synthesis of compound **2** required first protection of the amino group of the starting dehydroabietylamine and introduction of the hydroxy group in the aromatic ring by Friedel-Crafts acylation, Baeyer-Villiger oxidation, and overall hydrolysis of the resulting acetate-phthalimide. Subsequent deamination of **2** gave compound **1**. The ferruginol analogues **6**–**9** (phenols) were prepared from 12-hydroxydehydroabietylamine (**2**, 18-aminoferruginol), obtaining this material under an optimized protocol [16] and condensing it with appropriate substituted phthalic anhydrides using similar reported conditions [16]. Compounds **10**–**13** were prepared using reported conditions either from dehydroabietylamine through intermediate 18-(phthalimid-2-yl)ferruginol (**5**) [13] or from methyl dehydroabietate through intermediate methyl 12-hydroxyabieta-8,11,13-trien-18-oate (**14**) [17], which were readily obtained in four and three synthetic steps with 61% and 78% overall yield, respectively (Scheme 1). The approach relies on the ease with which phenols undergo oxidation. Thus, compounds **5** and **14** provided ortho-quinones **10** and **11**, which under mild reducing conditions led to the corresponding catechols **12** and **13**.

### 2.2. Biology

The compounds were systematically assessed through multiple biological testing platforms to establish their relative effectiveness and explore potential correlations between chemical structure and biological function. Firstly, their ability to inhibit the proliferation of three specific breast cancer linages (SUM149, MDA-MB231, MCF07) was evaluated in vitro by using the established CellTiter-Glo^®^ [18,19] cellular proliferation assay. The results are shown in Table 1 (IC_50_).

It was found that while most compounds exhibited biological activity, compounds **10** and **11** enhanced cytotoxic effects against triple-negative breast cancer cells in standard Cell-Titer-Glo viability assessments, suggesting a favorable differential between therapeutic efficacy and toxicity thresholds. The SUM149 model is emphasized due to its representation of the inflammatory and highly aggressive TNBC subtype.

Further evaluation of various promising compounds using breast cancer cells included analysis of colony-forming inhibition capabilities and investigations into their specific mechanistic pathways of action. Thus, the anchorage-dependent colony formation assay, also known as the clonogenic assay, was conducted. This method in cancer research evaluates the ability of single cells to grow into colonies when attached to a surface and can measure a cell’s ability to undergo “unlimited” division and form a colony (usually defined as ≥50 cells).

**Figure 2:**
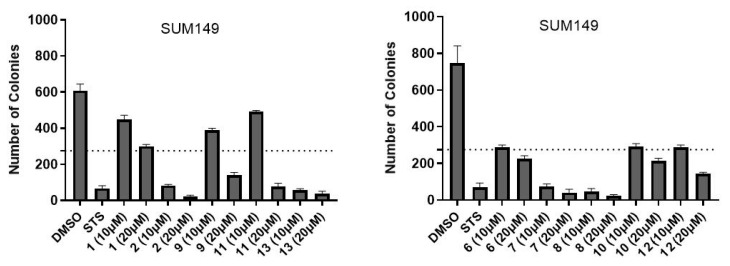
Quantification of colony formation assay (SUM149 cells) using ImageJ version 1.5.4 (NIH, Bethesda, MD, USA). STS: staurosporine. The dashed line marks the half-maximal inhibitory response.

**Figure 3:**
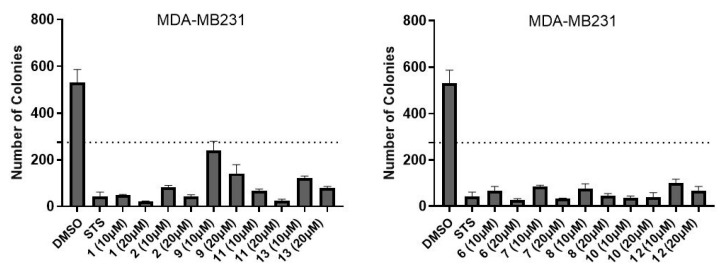
Quantification of colony formation assay (MDA-MB231 cells) using ImageJ version 1.5.4 (NIH, Bethesda, MD, USA). STS: staurosporine. The dashed line marks the half-maximal inhibitory response.

The clonogenic assay offers comprehensive insights into the compound’s overall efficacy and is regarded as the definitive method for assessing post-treatment reproductive cell death (both cytotoxic and cytostatic effects) [20]. The extended duration of the experiment simultaneously provides valuable data on the compounds’ stability over time, which are not detected in short-term viability assays. At a concentration of 20 μM, the compounds demonstrated clear efficacy in abolishing the colony-forming ability of SUM149 cells as depicted in Figure 2 (for image visualization, see Figures S1 and S2). The majority of compounds exhibited strong inhibitory effects even at 10 μM, with the notable exceptions of compounds **1**, and **10**–**11**—possibly attributable to the cellular mechanisms capable of hydrolyzing or neutralizing these particular agents. In the MDA-MB231 cellular model (Figure 3), the evaluated compounds successfully eliminated the cells’ capacity to establish colonies (for images, see Figures S3 and S4).

**Figure 4:**
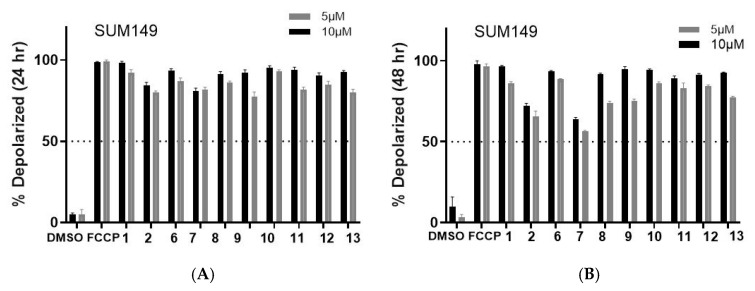
Relative TMRM mitochondrial % depolarization quantification of SUM149 cell model at two different time points for compound **1**–**2**, **6**–**13** at 5 or 10 μM. (**A**) after 24 h treatment. (**B**) 48 h treatment. DMSO (Vehicle, 0.1%). Positive control, FCCP (5 or 10 μM, treatment for 30 min). FCCP: carbonyl-cyanide-p-trifluoromethoxyphenylhydrazone. The dashed line marks the half-maximal inhibitory response.

**Figure 5:**
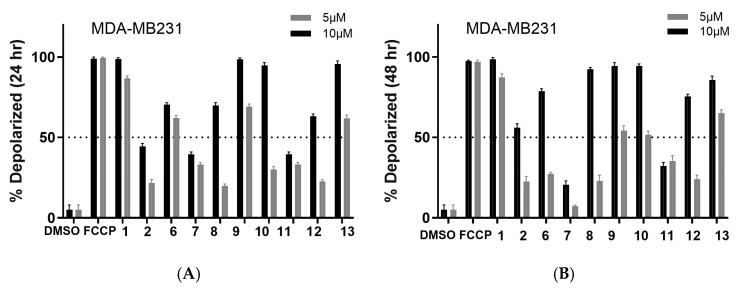
Relative TMRM mitochondrial % depolarization quantification of MDA-MB231 cell model at two different time points for compound **1**–**2**, **6**–**13** at 5 or 10 μM. (**A**) after 24 h treatment. (**B**) 48 h treatment. DMSO (Vehicle, 0.1%). Positive control, FCCP (5 or 10 μM, treatment for 30 min). FCCP: carbonyl-cyanide-p-trifluoromethoxyphenylhydrazone. The dashed line marks the half-maximal inhibitory response.

Finally, we evaluated the compounds’ potential to affect the mitochondrion. Mitochondrial depolarization is an early indicator of intrinsic apoptosis. Tetramethylrhodamine methyl ester perchlorate (TMRM) can detect this event before other apoptotic markers appear. Also, TMRM assays can measure metabolic reprogramming by assessing changes in mitochondrial potential. This assay can also provide further mechanistic studies by relaying off-target toxicity since it can capture unintended mitochondrial toxicity. It can also highlight if the cancer cell is likely to develop resistance to the compound since cancer cells that resist apoptosis often maintain mitochondrial potential despite treatment. TMRM helps to identify these resistant populations early in the drug discovery process. As shown in Figure 4 and Figure 5 (for images, see Figures S5 and S6), the data demonstrates that the compounds consistently induced mitochondrial membrane potential depolarization at both 5 and 10 µM concentrations, independent of exposure duration (24 or 48 h). However, SUM149 exhibited higher sensitivity compared to MDA-MB231, demonstrating a more pronounced response to all compounds except **1** and **9**. Furthermore, a reduction in potency was observed after 48 h treatment in both models, suggesting either compound degradation or cellular mechanisms actively expelling the compounds from the mitochondria. Nevertheless, the collective results suggest that these compounds are probably triggering cell death through apoptotic mechanisms.

**Figure 6:**
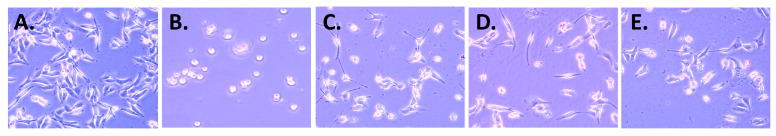
Brightfield microscopy images of MDA-MB231 breast cancer cells at 10× magnification following 24 h of treatment, showing morphological changes characteristic of early-stage apoptosis. (**A**) DMSO. (**B**) STS (5 μM). (**C**) Compound **1** (5 μM). (**D**) Compound **6** (5 μM). (**E**) Compound **10** (10 μM). STS: staurosporine.

Brightfield microscopy images of MDA-MB231 breast cancer cells are indicated in Figure 6 to highlight the morphological changes that occur upon treatment with compounds **1**, **6**, and **10** as representative compounds. Membrane blebbing, where the cell membrane forms irregular bulges or protrusions as the cytoskeleton detaches from the plasma membrane during the controlled cell death process, can be clearly observed with these compounds. The membrane blebbing is a key morphological feature of early apoptosis, where the cell membrane loses its normal structure and develops bubble-like protrusions as internal cellular components begin to fragment and the cell prepares for phagocytic clearance. Representative compounds demonstrated apoptotic activity. The precise mechanism of cell death for other compounds remained unclear.

### 2.3. In Silico Calculations

Calculations using computational tools are an important part of the drug development process. Using the SwissADME web server [21], through easily inserting the corresponding SMILES notation for each molecule from Chemdraw software 22.2.0, we can predict important molecular properties such as the Lipinski’s rule of five [22]. Representative results are displayed in Table 2 and Table S1, and complete results obtained from SwissADME are compiled in the Supplementary Materials (Figures S13–S25).

All compounds possess molecular weight (MW) values below 500 g/mol. The lipophilicity data by means of the computed consensus log P values ranged from 4.05 to 6.42, which caused the violation of the Lipinski’s rule log P < 5 for several compounds (Table 2). This indicates that most of these molecules may need reformulation to be orally active agents, which agrees with the water-solubility parameters giving values of moderate to poor solubility (Figures S13–S25). The pharmacokinetic data showed that high gastrointestinal absorption was observed for all compounds, while the blood–brain barrier (BBB) permeability potential was not predicted for all compounds (Table S1). Half of the compounds showed the potential to be substrates for P-gp. A lack of inhibitory potential against cytochrome P450 (CYP) isoforms was observed for most compounds, particularly for CYP1A2 and CYP2D6 isoforms. Interestingly, compound 2 was predicted to potentially inhibit only CYP2D6 out of the five isoforms calculated (Table S1).

## 3. Discussion

The research on the interesting family of naturally occurring abietane diterpenoids found in many plants has attracted significant attention from the natural products, synthetic, medicinal chemistry, and pharmacological communities due to their diverse structures and wide range of biological properties [5,6,23,24,25,26,27].

Over the last decade, we have contributed to this highly dynamic field of research, particularly in the semi-synthesis of natural abietanes, derivatives, or analogues and the study of their biological activities. Our objective was consistently focused on identifying compounds with improved therapeutic profiles while simultaneously validating the structural assignments during the isolation process, particularly when working with natural abietane scaffolds as established targets. During our investigations, we developed a scalable synthesis of (+)-ferruginol (**1**), through the intermediate 18-aminoferruginol (**2**) starting from commercial (+)-dehydroabietylamine (Scheme 1) [14]. This led to the discovery of 18-(phthalimid-2-yl)ferruginol (**5**) as a broad-spectrum antiviral agent [28], encouraging the efforts towards the synthesis of ferruginol analogues and their biological profiling. The previous literature demonstrating the biological activity of ferruginol (**1**), tanshinone IIA (**3**), and carnosol (**4**) has informed our research direction [13,17]. We found that compounds **5**, **10**, **11**, and **12** displayed the highest efficacy inhibiting the proliferation of several breast cancer cell lines (IC_50_ = 1.3–13 μM and therapeutic index range 6–43) (Table 1) [13], thus encouraging further studies herein presented. This compelling finding highlights the crucial importance of natural product derivatization in identifying potent anticancer compounds with enhanced biological activity and availability. Compounds **10** and **11**, structurally, are characterized by an orthoquinone functionality at C-11 and C-12 which surely is responsible for their antiproliferative effects. These results aligned well with extensive research on the cytotoxicity of abietane-type diterpenes containing a quinone moiety [29,30]. Ferruginol analogues **6** and **7**, containing an atom of fluorine in the phthalimide, were more potent than the parent ferruginol (**1**) with moderate potency (Table 1). Similarly, the new phthalimide analogues **8** and **9** with methoxy substituents were more potent than ferruginol (**1**), except compound **8** for the MDA-MB231 line, displaying similar activities to that of **5**–**7**, generally (Table 1).

From a structure–activity perspective, this focused compound library (**1**, **2**, **5**–**13**) shows that heteroatom enrichment around the C ring of the abietylamine core selectively boosts anti-TNBC potency without significantly affecting ER-positive cell viability. The contrast between compounds **5** and **10** provides a representative example of the addition of one oxygen atom. Furthermore, the phthalimide substituent on the side chain plays a key role in improving solubility parameters and increasing structural bulk, evident from the comparison of compounds **6**–**9** relative to compound **1**. Combining these changes in the molecules, improved solubility, and likely stability to support better target engagement, more detailed mechanistic work is necessary to validate this hypothesis.

## 4. Materials and Methods

### 4.1. Chemistry: General Experimental Procedures

Specific rotation was measured using a 10 cm cell in a Jasco P-2000 polarimeter (JASCO Corporation, Tokyo, Japan) in DCM. NMR data were collected on a 400 MHz spectrometer (Bruker, Billerica, MA, USA). All spectra were recorded in CDCl_3_ as solvent. Reactions were monitored by TLC using Merck silica gel 60 F254 (0.25 mm-thick) plates (Merck, Rahway, NJ, USA). Compounds on TLC plates were visualized under UV light at 254 nm and by immersion in a 10% sulfuric acid solution and heating with a heat gun. Purifications were performed by using flash chromatography on Merck silica gel (230–400 mesh). Commercial reagent grade solvents and chemicals were used as purchased. Combined organic extracts were washed with brine, dried over anhydrous MgSO_4_, filtered, and concentrated under reduced pressure.

(+)-Dehydroabietylamine (ca. 90%) and (−)-abietic acid >70% were purchased from from TCI Europe (Zwijndrecht, Belgium). Carbon numbering of compounds matched that of natural products (abietanes skeleton). All known compounds prepared in this work displayed spectroscopic data in agreement with the reported data [13,15,16,17]. The purity of the final compound was 95% or higher. Copies of NMR spectra for new compounds **8**, **9** are available in the Supplementary Information.

### 4.2. Synthesis

12-Hydroxy-N,N-(3-methoxyphthaloyl)dehydroabietylamine (**8**)

A mixture of 3-methoxyphthalic anhydride (356 mg, 2.0 mmol) and 12-hydroxydehydroabietylamine [16] (300 mg, 0.99 mmol) in acetic acid (4.0 mL) was heated at reflux for 3 h, then cooled to RT, and 30 mL of water were added. The resulting solid was filtered off, washed with water, dried under vacuum (590 mg) and purified by column chromatography eluting with *n*-hexane-ethyl acetate 6:4 to give compound **8** (288 mg, 62%) as a pale oil which became a light pale solid after trituration with *n*-hexane-dichloromethane: mp = 122–125 °C; [α]^20^_D_ −22.6 (c 1.0, CH_2_Cl_2_); ^1^H NMR (400 MHz, CDCl_3_) *δ* 7.64 (1H, dd, *J* = 8.4, 7.2), 7.40 (1H, d, *J* = 7.2), 7.17 (1H, dd, *J* = 8.4), 6.85 (1H, s), 6.60 (1H, s), 4.85 (1H, br s, OH), 3.99 (3H, s, CO_2_*CH*_3_), 3.63 (1H, d, *J* = 13.6), 3.48 (1H, d, *J* = 13.6), 3.11 (1H, sept., *J* = 6.8), 2.94–2.89 (2H, m), 2.25–2.18 (1H, m), 2.10 (1H, dd, *J* = 12.8, 2.0), 1.81–1.59 (3H, m), 1.50–1.25 (4H, m), 1.23 (3H, d, *J* = 6.8), 1.20 (3H, d, *J* = 6.8), 1.19 (3H, s), 1.02 (3H, s); ^13^C NMR (100 MHz, CDCl_3_) *δ*_C_ 169.1 (s), 168.0 (s), 156.4 (s), 150.6 (s), 148.2 (s), 136.0 (d), 134.1 (s), 131.5 (s), 127.2 (s), 126.7 (d), 117.2 (s), 117.2 (d), 115.4 (d), 110.5 (d), 56.2 (q), 48.7 (t), 45.1 (d), 39.3 (s), 38.0 (t), 37.5 (s), 36.8 (t), 29.3 (t), 26.7 (d), 25.7 (q), 22.6 (q), 22.5 (q), 19.5 (t), 19.1 (q), 18.4 (t); HRMS (ESI) m/z 460.2471 [M-H]^–^, calcd for C_29_H_34_NO_4_: 460.2488; Anal. calcd. for C_29_H_35_NO_4_: C, 75.5; H, 7.6; N, 3.0; Found: C, 75.2; H, 7.2; N, 2.6.

12-Hydroxy-N,N-(4-methoxyphthaloyl)dehydroabietylamine (**9**)

A mixture of 4-methoxyphthalic anhydride (356 mg, 2.0 mmol) and 12-hydroxydehydroabietylamine [16] (300 mg, 0.99 mmol) in acetic acid (4.0 mL) was heated at reflux for 3 h, then cooled to RT, and 30 mL of water were added. The resulting solid was filtered off, washed with water, dried under vacuum (170 mg) and purified by column chromatography eluting with *n*-hexane-ethyl acetate 6:4 to give compound **9** (80 mg, 18%) as a pale oil which became a light pale solid after trituration with *n*-hexane-dichloromethane: mp = 115–118 °C; [α]^20^_D_ −24.0 (c 1.0, CH_2_Cl_2_); ^1^H NMR (400 MHz, CDCl_3_) *δ* 7.70 (1H, d, *J* = 8.0), 7.28 (1H, d, *J* = 2.4), 7.12 (1H, dd, *J* = 8.4, 2.4), 6.85 (1H, s), 6.59 (1H, s), 3.90 (3H, s, CO_2_*CH*_3_), 3.65 (1H, d, *J* = 14.0), 3.47 (1H, d, *J* = 14.0), 3.10 (1H, sept., *J* = 6.8), 2.92–2.89 (2H, m), 2.26–2.08 (2H, m), 1.82–1.61 (4H, m), 1.50–1.25 (3H, m), 1.23 (3H, d, *J* = 6.8), 1.21 (3H, d, *J* = 6.8), 1.21 (3H, s), 1.03 (3H, s); ^13^C NMR (100 MHz, CDCl_3_) *δ*_C_ 169.2 (s), 169.1 (s), 164.6 (s), 150.6 (s), 148.2 (s), 134.5 (s), 131.5 (s), 127.3 (s), 126.8 (d), 124.9 (d), 123.9 (s), 119.8 (d), 110.5 (d), 107.8 (d), 56.0 (q), 48.8 (t), 45.0 (d), 39.4 (s), 38.1 (t), 37.5 (s), 36.8 (t), 29.3 (t), 26.7 (d), 25.7 (q), 22.7 (q), 22.6 (q), 19.5 (t), 19.1 (q), 18.5 (t); HRMS (ESI) *m*/*z* 460.0275 [M-H]^–^, calcd for C_29_H_34_NO_4_: 460.2488; Anal. calcd. for C_29_H_35_NO_4_: C, 75.5; H, 7.6; N, 3.0; Found: C, 75.1; H, 7.7; N, 2.8.

### 4.3. Cells

Human cell lines were incubated at 37 °C in a 5% CO_2_ atmosphere and maintained under sterile conditions [31]. Cells were tested for Mycoplasma (Lonza, Alpharetta, GA, USA) using the manufacturer’s conditions prior to experiments. Cell lines were purchased from American Type Culture Collection (ATCC, Manassas, VA, USA), and SUM149 was obtained from Asterand (Detroit, MI, USA) and cultured without antibiotics unless stated. Cells were cultured in Eagle’s Minimum Essential Medium (EMEM), DMEM, or F12 from ATCC supplemented with 10% fetal bovine serum (FBS, Hyclone, Logan, UT, USA) and 1% GlutaMAX. Human BJ fibroblast cells (ATCC, CRL-2522) were supplemented with 10% fetal bovine serum (FBS, Hyclone, Logan, UT, USA) and 1% GlutaMAX. BJ cells were cultured in EMEM media, and breast cancer cells (MDA-MB-231, MCF07) were cultured in DMEM. SUM149 was cultured in Ham’s F12 supplemented with 1% GlutaMAX,1% Pen/Step, 2 μM cortisol, and 1 ug/mL insulin. Cells were grown to 80% confluence densities as recommended by ATCC.

### 4.4. Cell Viabiliy (CellTiterGlo) Assay

Cytotoxicity evaluation was performed using the CellTiter-Glo Luminescent Cell Viability Assay kit (G7570, Promega, Madison, WI, USA), according to the manufacturer’s instructions. Briefly, the cell concentrations used were experimentally determined to ensure logarithmic growth during the 72 h duration of the experiment and avoid adverse effects on cell growth by DMSO exposure. To proceed, 1 × 10^5^–3 × 10^5^ cells/well were seeded in 96-well white flat-bottomed plates (#3610 Corning, Corning, NY, USA) in 100 mL/well. The plates were incubated at 37 °C in 5% CO_2_ for 24 h before drugging. Test compounds (10 mM in DMSO) in eight 2-fold serial dilutions were dispensed to assay plates. The final concentration of DMSO was 0.3% (*v*/*v*) in each well. The positive controls included staurosporine (10 μM) and/or gambogic acid (10 μM). The plates were incubated for 72 h at 37 °C in 5% CO_2_, then quenched with CellTiter-Glo^®^ (Promega, Madison, WI, USA, 50 uL/well), centrifuged at 1000 rpm for 1 min, and incubated at RT for 20 min. Luminescence was recorded with a plate reader (CLARIOstar Plus, BMG LabTech, Ortenberg, Germany). The mean luminescence of each experimental treatment group was normalized as a percentage of the mean intensity of untreated controls. EC_50_ values were calculated from dose-response curve-fitting via non-linear regression using GraphPad Prism 9.0 (GraphPad Software, San Diego, CA, USA). A therapeutic index (TI) between normal and tumor cell lines can be determined (EC_50_ non-neoplastic cell line BJ)/(EC_50_ cancer cell line).

### 4.5. Cell Morphology

Representative images were collected using 10× objective with a Leica DMi1 Microscope (Leica microsystems, Wetzlar, Germany). MDA-MB231 cells were imaged with a vehicle as the negative control, staurosporine as the positive control (2 μM), or compounds with results shown in representative images for 24 h.

### 4.6. Colony Formation Assay

MDA-MB-231 (0.8 × 10^6^ cells/well) and SUM-149 cells (1.5 × 10^6^ cells/well) were plated on a 6-well plate. Two days later, cell media was changed to 5% FBS, and cells were incubated at 37 °C in a 5% CO_2_ atmosphere for 1 h before the vehicle (0.1% DMSO) or compound (5–10μM) was added. After 72 h of treatment, cells were trypsinized and reseeded at 800 cells/mL (MDA-MB231) and 1200 cells/mL (SUM149) per well in 6-well plates. After 10 days of culture, the media was removed from the wells and washed once with cold PBS. The colonies were stained with 5 mL of 1% Crystal Violet for 30 min. The dishes were fixed by methanol, rinsed with water three times, and air-dried, and the colonies were counted using ImageJ version 1.5.4 (NIH, Bethesda, MD, USA).

### 4.7. Tetramethylrhodamine Methyl Ester Perchlorate (TMRM) Assay

The TMRM assay was performed as described [19]. Briefly, SUM-149 cells (5 × 10^5^ cells/well) or MDA-MB231 cells (2 × 10^5^ cells/well) were seeded in 6-well plates for 24 h at 37 °C, 5% CO_2_. Cells were then treated with either the vehicle (DMSO 0.2%) or 20 μM FCCP (24 h). Processing was carried out using the Image-iT TMRM Reagent (Invitrogen, No. I34361, Carlsbad, CA, USA). FCCP was used as the positive control and was added for 10 min prior to TMRM staining. The cell culture medium was removed, and cells were washed with phosphate buffered saline (PBS), trypsinized, or resuspended in PBS supplemented with 1% FBS and analyzed by flow cytometry.

### 4.8. Statistical Analysis

Statistical analysis of data was performed using GraphPad Prism (Version 10.4.1 San Diego, CA, USA) and/or Microsoft Excel software (Office 2010, Microsoft Corp., Redmond, WA, USA). The statistical methods used were repeated-measures analysis of variance and Tukey’s test for paired data when appropriate.

### 4.9. ADMET and Drug-Likeness Analysis

The prediction of absorption, distribution, metabolism, excretion, and toxicity (ADMET) parameters and drug-like properties of the compounds was performed using the SwissADME server (http://www.swissadme.ch/, accessed on 6 February 2025) [21]. Key physicochemical parameters calculated encompassed molecular weight (MW), atom counts, and polar surface area (PSA). Lipophilicity was evaluated via iLOGP, XLOGP3, WLOGP, MLOGP, and SILICOS-IT models to generate log P octanol/water (log Po/w) values and consensus log P data. Aqueous solubility (log S) values were predicted through ESOL, Ali, and SILICOS-IT models. The pharmacokinetic descriptors examined included gastrointestinal absorption (GI), blood–brain barrier (BBB) permeability, skin permeability (log Kp), and the substrate potential of P-glycoprotein (P-gp), as well as the inhibition of five major cytochrome P450 isoforms (CYP1A2, CYP2C19, CYP2C9, CYP2D6, and CYP3A4). Compound drug-likeness was assessed through established criteria, including Lipinski, Ghose, Veber, Egan, and Muegge filters.

## 5. Conclusions

Our studies demonstrate that these compounds show strong efficacy against breast cancer subtypes. While the exact mechanisms underlying their activity remain to be fully elucidated, these results indicate that these specialized compounds warrant additional research, particularly to uncover the basis for their notable selectivity toward specific cancer subtypes. For instance, the diketone system in compounds **10**–**11** exhibited notable effectiveness against SUM149 cell line. While compounds **11**–**12** exhibited comparable bioactivity profiles across the tested cell lines, compound **13** showed minimal activity.

The compounds exhibit a consistent bioactivity pattern between cytotoxicity and functional assays like colony formation, further confirming their potential in solid tumor treatment. Examination of the abietane molecular scaffold reveals it provides a robust structural platform with nuanced features that influence biological activity. More significant structural modifications, such as those seen in compounds **6**–**8**, which feature a less oxidized C ring and incorporate a phthalimide group on the A ring, consistently demonstrate pronounced biological effects across multiple experimental systems—from cell viability assessments to colony formation inhibition studies in various cancer cell lines.

## Figures and Tables

**Table 1:** Cytotoxicity (IC_50_ in µM) of semisynthetic abietanes **1**–**2**, and **5**–**13** against three cell lines isolated from breast cancer after 72 h of incubation ^a^.

Compound	SUM149	TI	MDA-MB231	TI	MCF07	TI	BJ
**1** ^b^	>50	>1	8.3 ± 1.4	>6	19.0 ± 1.5	>3	>50
**2** ^b^	4.4 ± 0.3	17	5.1 ± 0.6	15	10.0 ± 1.5	7	75.0 ± 6.2
**5** ^b^	4.4 ± 0.2	17	8.2 ± 0.5	9	13.0 ± 0.6	6	74.8 ± 5.3
**6**	4.8 ± 0.2	13	9.4 ± 0.9	7	5.4 ± 0.6	12	63.5 ± 6.3
**7**	4.3 ± 0.1	>12	7.6 ± 0.2	>7	7.0 ± 0.6	>7	>50
**8**	5.3 ± 0.1	12	54.6 ± 5.0	≈1	11.0 ± 1.4	6	61.2 ± 5.4
**9**	4.3 ± 0.2	14	8.2 ± 0.8	7	12.0 ± 1.2	5	58.9 ± 5.7
**10** ^b^	1.5 ± 0.4	24	2.8 ± 0.4	13	2.3 ± 1.1	15	35.4 ± 5.0
**11** ^b^	1.3 ± 0.6	43	3.9 ± 0.8	15	8.3 ± 0.9	7	56.5 ± 6.1
**12** ^b^	1.8 ± 0.2	18	2.8 ± 0.2	12	4.6 ± 0.6	7	32.5 ± 5.0
**13** ^b^	>50	≈1	9.4 ± 0.4	6	>50	≈1	57.6 ± 3.0

^a^ Cells were treated with compounds for 72 h. Cytotoxicity was determined as the IC_50_ values calculated from the percentage of viable cells remaining at 72 h, measured with CellTiter-Glo reagent. The IC_50_ values are indicated as the mean ± SD of at least three independent experiments. TI: Therapeutic index = (EC50 non-neoplastic human BJ fibroblasts cell line)⁄(EC50 cancer cell line); ^b^ Values taken from reference [13].

**Table 2:** Physicochemical and molecular properties (drug-likeness) by using SwissADME Online for compounds **1**–**13** ^a,b^.

Compound	Consensus Log P	MW	n-HBA	n-HBD	TPSA	Lipinski’s Violation
**1**	5.50	286.45	1	1	20.23	1
**2**	4.65	301.47	2	2	46.25	0
**3**	5.37	294.34	3	0	47.28	1
**4**	4.05	330.42	4	2	66.76	0
**5**	6.00	431.57	3	1	57.61	1
**6**	6.42	449.56	4	1	57.61	1
**7**	6.42	449.56	4	1	57.61	1
**8**	6.08	461.59	4	1	66.84	1
**9**	6.08	461.59	4	1	66.84	1
**10**	5.51	445.55	4	0	71.52	1
**11**	4.48	344.44	4	0	60.44	0
**12**	5.52	447.57	4	2	77.84	1
**13**	4.50	346.46	4	2	66.76	0
Rule of five	not >5	<500	not >10	not >5		1 violation allowed

^a^ Values were calculated using SwissADME webpage (http://www.swissadme.ch, accessed on 6 February 2025). ^b^ consensus Log P = consensus log partition coefficient; MW = Molecular weight; n-HBA = number of hydrogen bond accepting groups; n-HBD = number of hydrogen bond donating groups; TPSA = Total polar surface area.

## Data Availability

The data are available in the manuscript and the Supporting Information of this article.

## Supplementary Materials

The following supporting information can be downloaded at: https://www.mdpi.com/article/10.3390/molecules30122529/s1, Figures S1–S4: Images of colony formation assays; Figures S5 and S6: Images of TMRM assays; Figures S7–S12: copies of ^1^H, ^13^C, and DEPT NMR spectra of **8**, **9**; Figures S13–S25: ADMET data of compounds **1**–**13**; Table S1, page S24: pharmacokinetic parameters of compounds **1**–**13**.
