# Supplementary material for: In Vitro Effect of Ferruginol, Tanshinone, and Carnosol Analogues on the Proliferation of Three Breast Cancer Cell Lines
Source: Molecules. 2025 Jun 10;30(12):2529. doi: 10.3390/molecules30122529 (PMC12195677; doi:10.3390/molecules30122529)
Supplement: Supplementary file 1 [file molecules-30-02529-s001.zip › molecules-3685214-supplementary.pdf]

Supplementary Information

In Vitro Effect of Ferruginol, Tanshinone, and Carnosol Analogues on the Proliferation of Three Breast Cancer Cell Lines

Miguel A. González-Cardenete [1,*], William E. Mendoza-Hernández [1], Sydney L. Lawson [2], Fatima Rivas [2,*]

[1] Instituto de Tecnología Química, Universitat Politècnica de València-Consejo Superior de Investigaciones Científicas, Avda. de los Naranjos s/n, 46022 Valencia, Spain; wemenher@itq.upv.es
[2] Department of Chemistry, Louisiana State University, 133 Choppin Hall, Baton Rouge, LA 70803, USA

* Correspondence: migoncar@itq.upv.es (M.A.G.-C.); frivas@lsu.edu (F.R.)

Contents

• Images of colony formation assays……………………………………………Figures S1-S4

• Images of TMRM assays………………………………………………………Figures S5-S6

• Copies of ^{1}H NMR , ^{13}C NMR and DEPT spectra for new compounds **8**, **9**:

-^{1}H NMR spectrum of **8**…………….………..………………………………Figure S7

-^{13}C NMR spectrum of **8**…………..…..…………………………………………Figure S8

-DEPT135 spectrum of **8**……………………………………………………Figure S9

-^{1}H NMR spectrum of **9**…………….………..………………………………Figure S10

-^{13}C NMR spectrum of **9**…………..…..…………………………………………Figure S11

-DEPT135 spectrum of **9**……………………………………………………Figure S12

• Copies of ADMET calculations from SWISSADME web server for **1-13**…Figures S13-S25

• Table S1. Predicted pharmacokinetics parameters for **1-13**……………………page S24

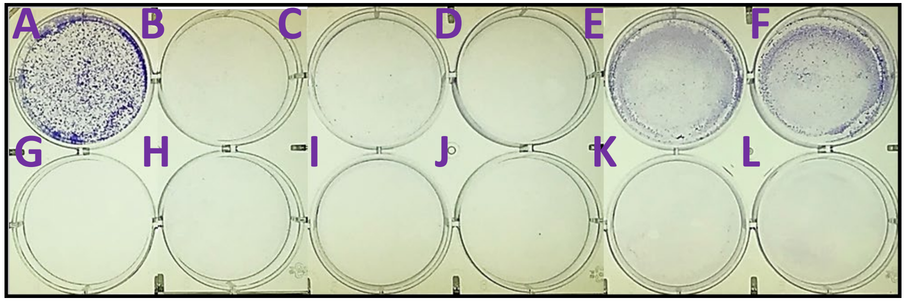

Figure S1. Representative images of colony formation assay (SUM149 cells). **A. DMSO**, **B. 9** (10 μM), **C. 13** (10 μM), **D. 2** (10 μM), **E. 1** (10 μM), **F. 11** (10 μM), **G.** STS (5 μM), **H. 9** (20 μM), **I. 13** (20 μM), **J. 2** (20 μM), **K. 1** (20 μM), **L. 11** (20 μM). STS: staurosporine.

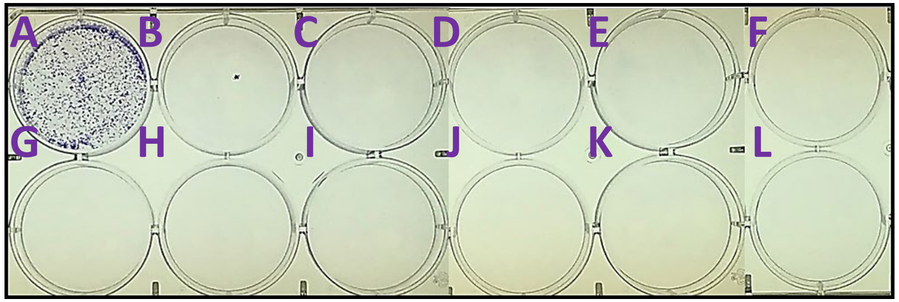

Figure S2. Representative images of colony formation assay (SUM149 cells). **A.** DMSO, **B. 10** (10 μM), **C. 7** (10 μM), **D. 6** (10 μM), **E. 8** (10 μM), **F. 12** (10 μM), **G.** STS (5 μM), **H. 10** (20 μM), **I. 7** (20 μM), **J. 6** (20 μM), **K. 8** (20 μM), **L. 12** (20 μM). STS: staurosporine.

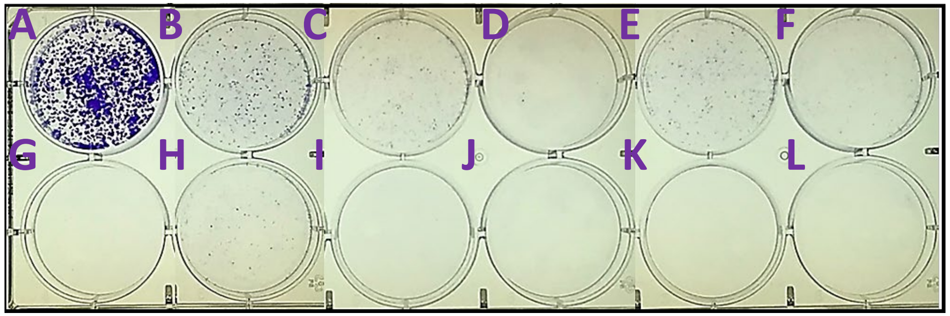

Figure S3. Representative images of colony formation assay (MDA-MB231 cells). **A. DMSO**, **B. 9** (10 μM), **C. 13** (10 μM), **D. 2** (10 μM), **E. 1** (10 μM), **F. 11** (10 μM), **G.** STS (5 μM), **H. 9** (20 μM), **I. 13** (20 μM), **J. 2** (20 μM), **K. 1** (20 μM), **L. 11** (20 μM). STS: staurosporine.

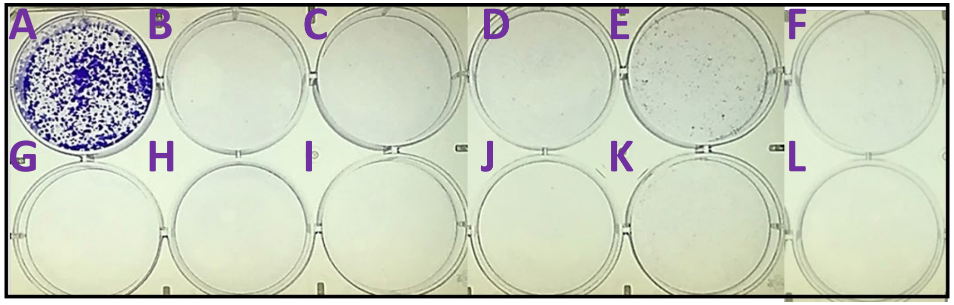

Figure S4. Representative images of colony formation assay (MDA-MB231 cells). **A.** DMSO, **B. 10** (10 μM), **C. 7** (10 μM), **D. 6** (10 μM), **E. 8** (10 μM), **F. 12** (10 μM), **G.** STS (5 μM), **H. 10** (20 μM), **I. 7** (20 μM), **J. 6** (20 μM), **K. 8** (20 μM), **L. 12** (20 μM). STS: staurosporine.

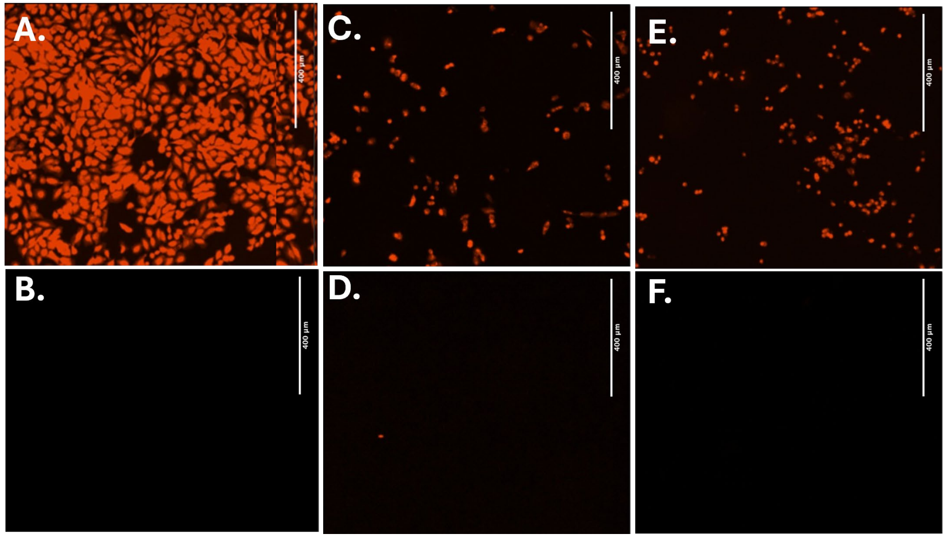

Figure S5. Fluorescence microscopy image showing TMRM-stained SUM149 breast cancer cells at 4X magnification after 24 h of treatment, demonstrating significant mitochondrial membrane potential loss in treated cells with increasing concentration-dependent effects. **A.** DMSO. **B.** FCCP (10 µM, after 30 min treatment). **C.** Compound **1** (5 µM) **D.** Compound **1** (10 µM) **E.** Compound **6** (5 µM) **F.** Compound **6** (10 µM). FCCP: carbonyl-cyanide-p-trifluoromethoxyphenylhydrazone.

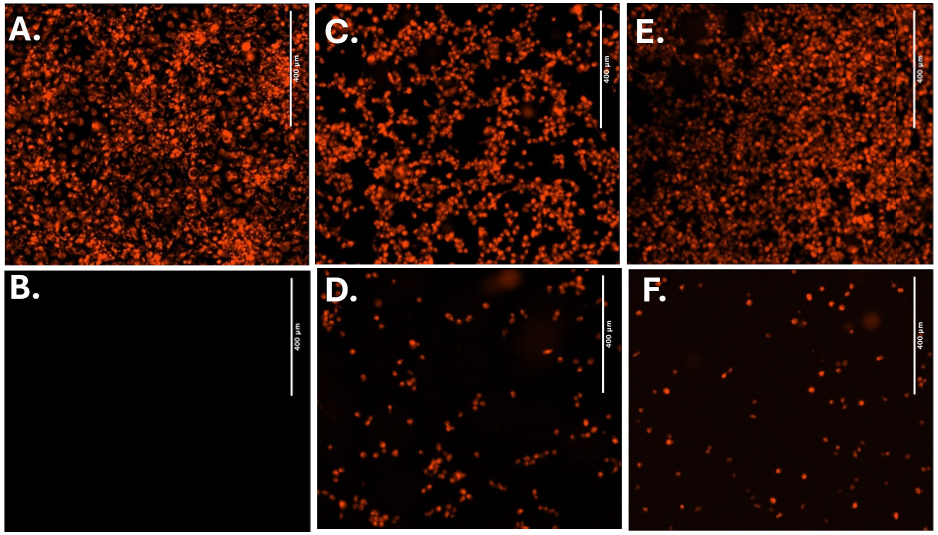

Figure S6. Fluorescence microscopy image showing TMRM-stained MDA-MB-231 breast cancer cells at 4X magnification after 24 h of treatment, demonstrating significant mitochondrial membrane potential loss in treated cells with increasing concentration-dependent effects. **A.** DMSO. **B.** FCCP (10 µM, 30 min). **C.** Compound **1** (5 µM) **D.** Compound **1** (10 µM) **E.** Compound **6** (5 µM) **F.** Compound **6** (10 µM). FCCP: carbonyl-cyanide-p-trifluoromethoxyphenylhydrazone.

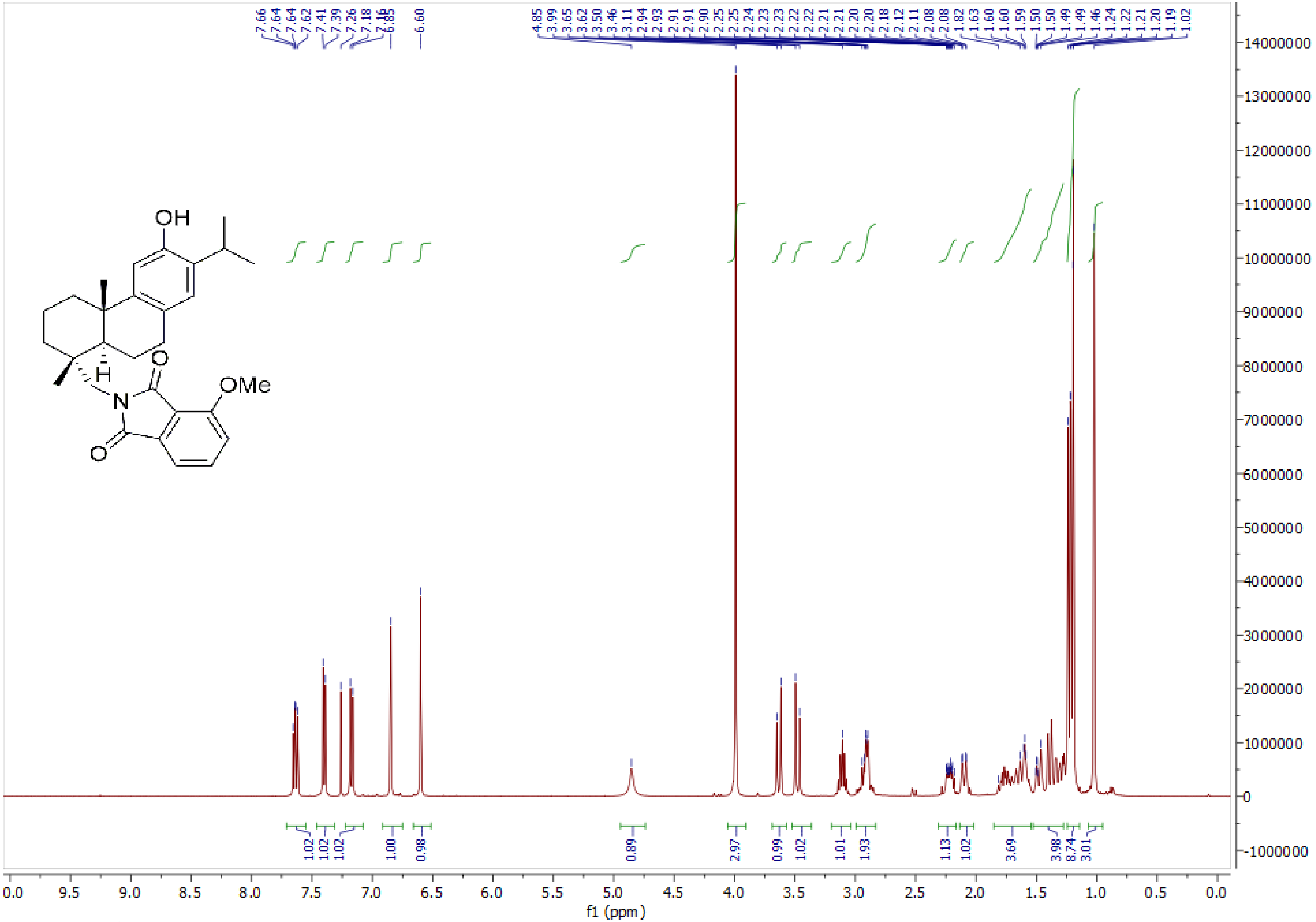

Figure S7. ^{1}H NMR spectrum (400 MHz, $CDCl_3$) of 12-Hydroxy-N,N-(3-methoxyphthaloyl)dehydroabietylamine (**8**).

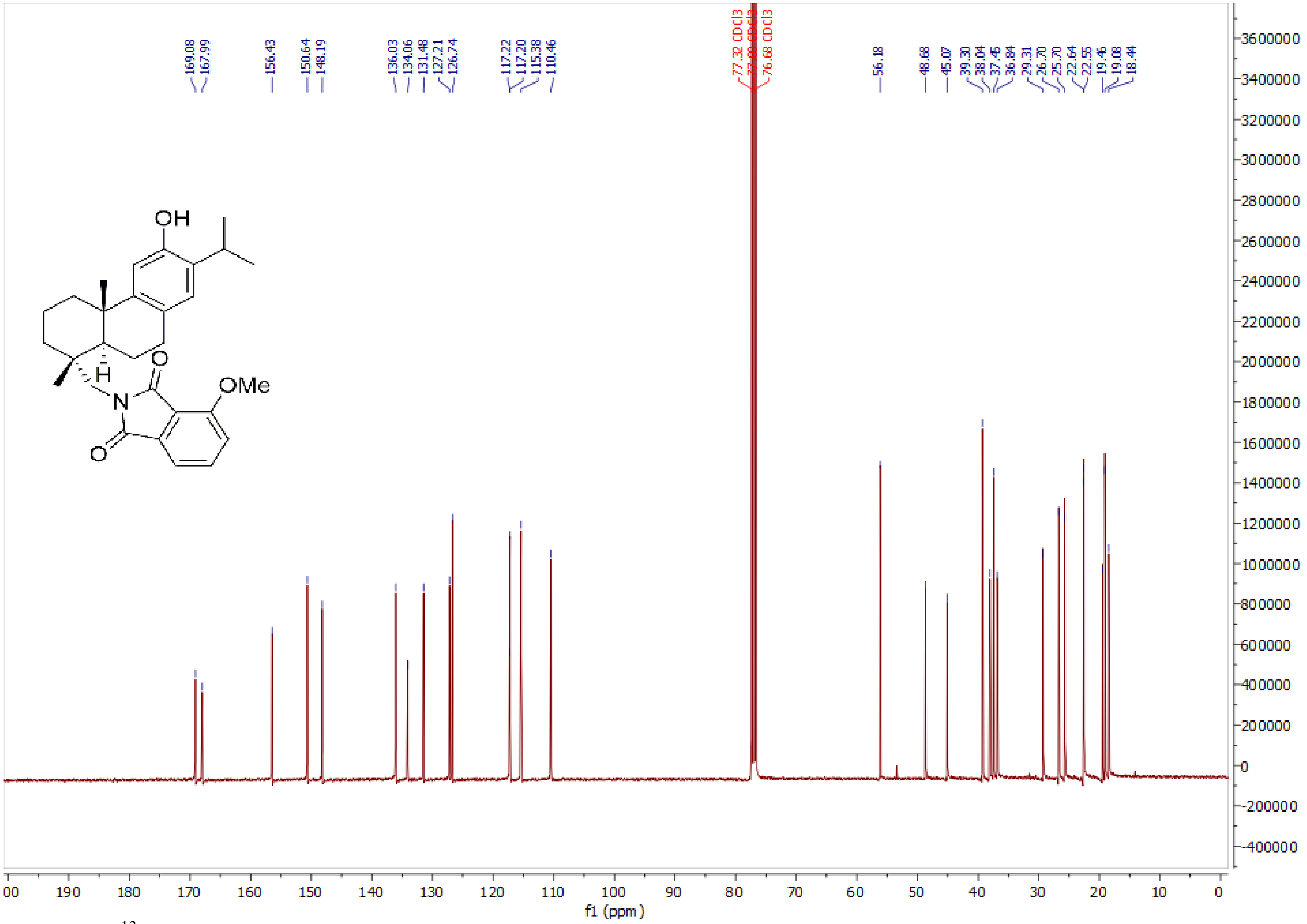

Figure S8. ^{13}C NMR spectrum (100 MHz, $CDCl_3$) of 12-Hydroxy-N,N-(3-methoxyphthaloyl)dehydroabietylamine (**8**).

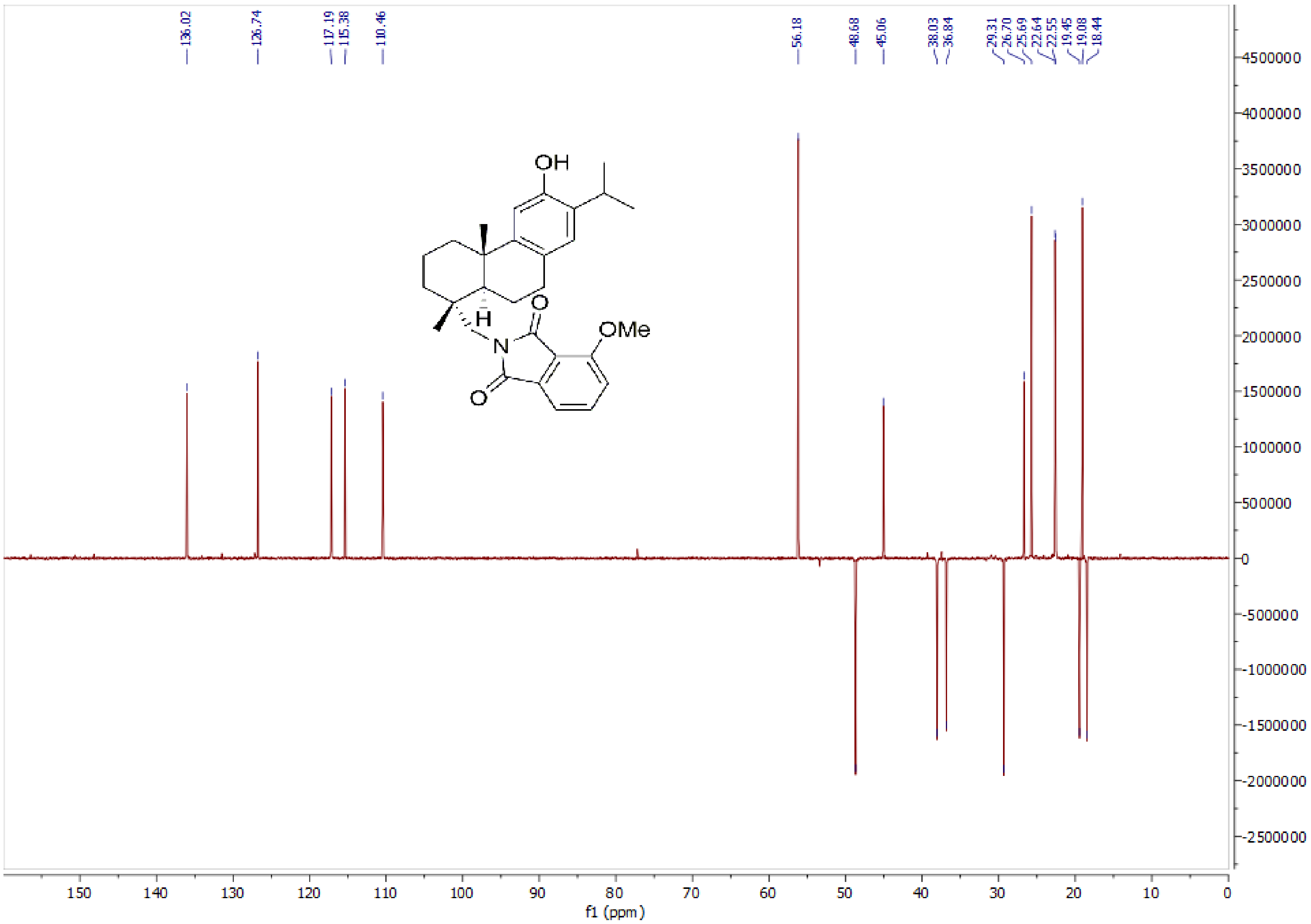

Figure S9. DEPT135 spectrum (100 MHz, $CDCl_3$) of 12-Hydroxy-N,N-(3-methoxyphthaloyl)dehydroabietylamine (**8**).

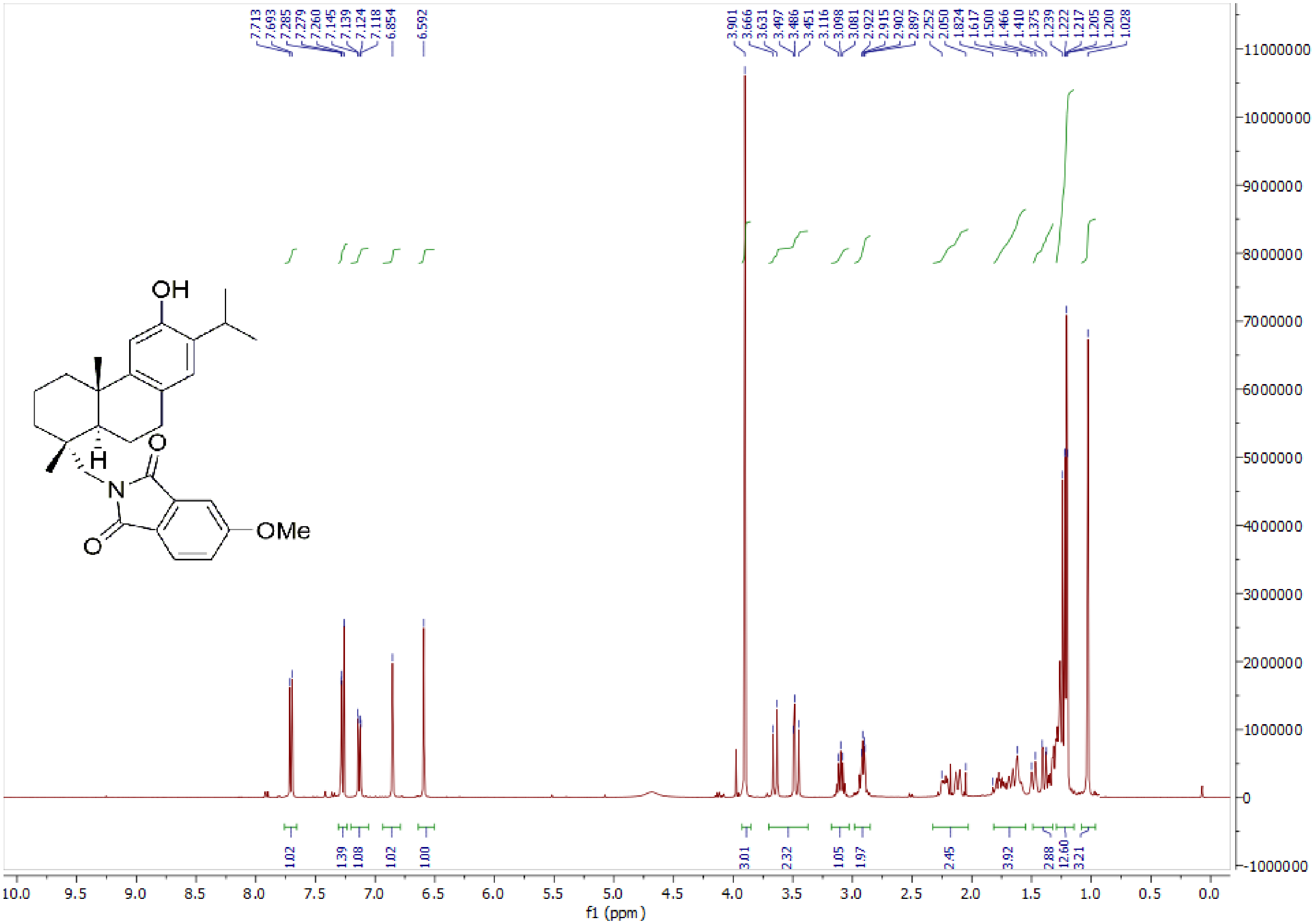

Figure S10. ^{1}H NMR spectrum (400 MHz, $CDCl_3$) of 12-Hydroxy-N,N-(4-methoxyphthaloyl)dehydroabietylamine (**9**).

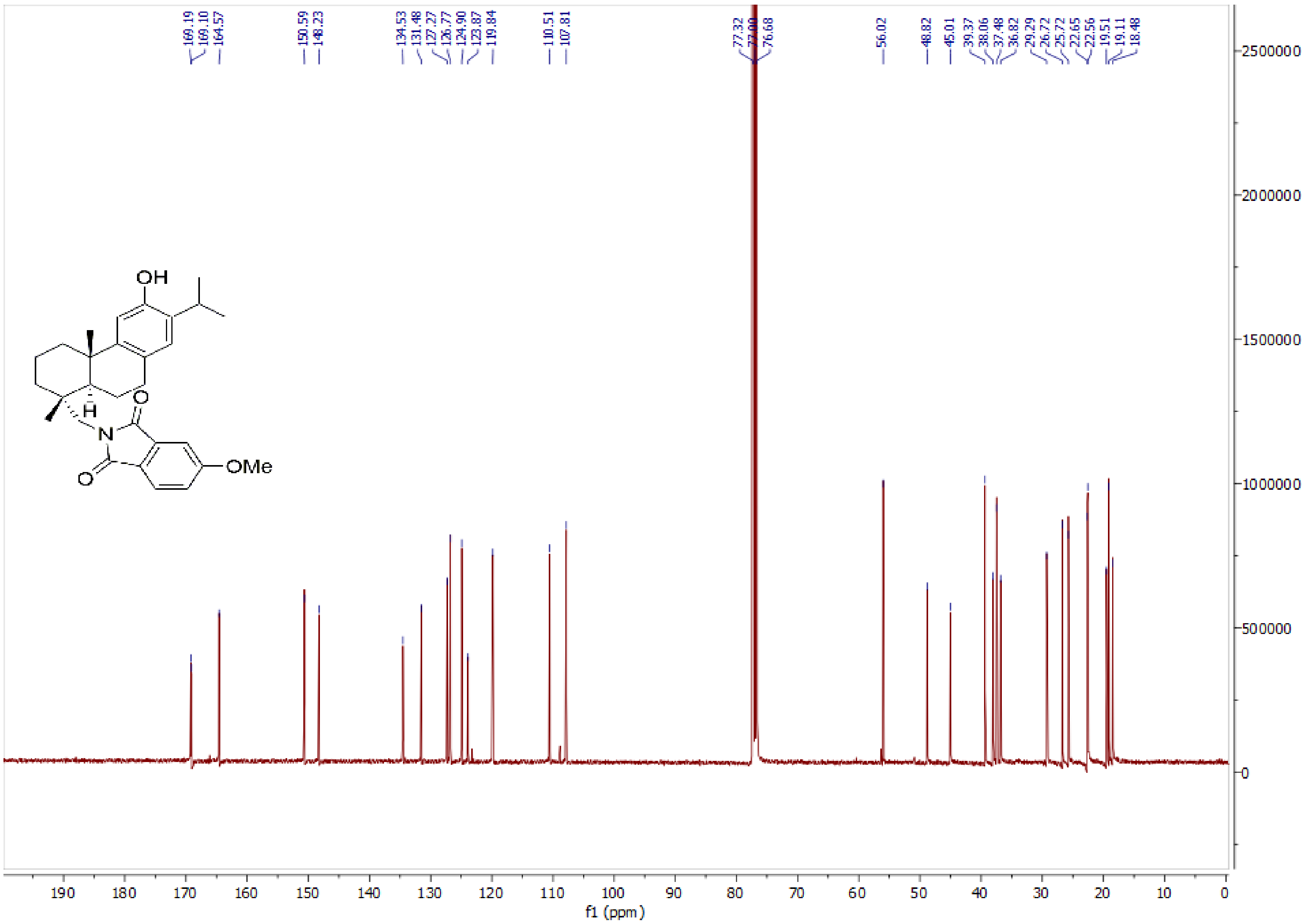

Figure S11. ^{13}C NMR spectrum (100 MHz, $CDCl_3$) of 12-Hydroxy-N,N-(4-methoxyphthaloyl)dehydroabietylamine (**9**).

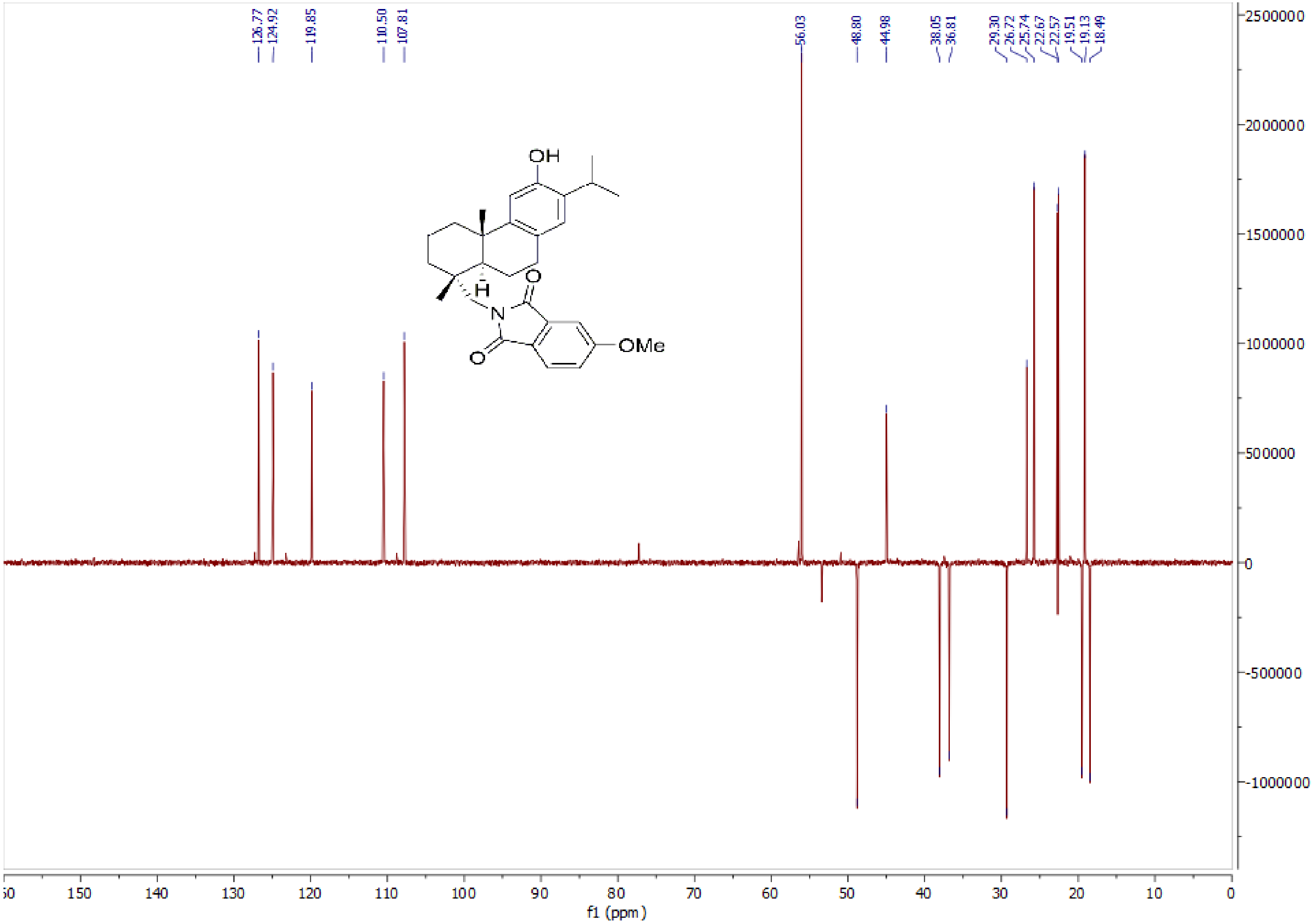

Figure S12. DEPT135 spectrum (100 MHz, $CDCl_3$) of 12-Hydroxy-N,N-(4-methoxyphthaloyl)dehydroabietylamine (**9**).

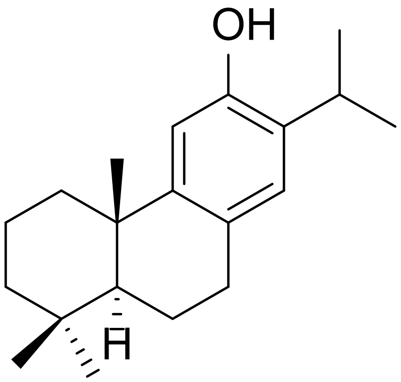

1

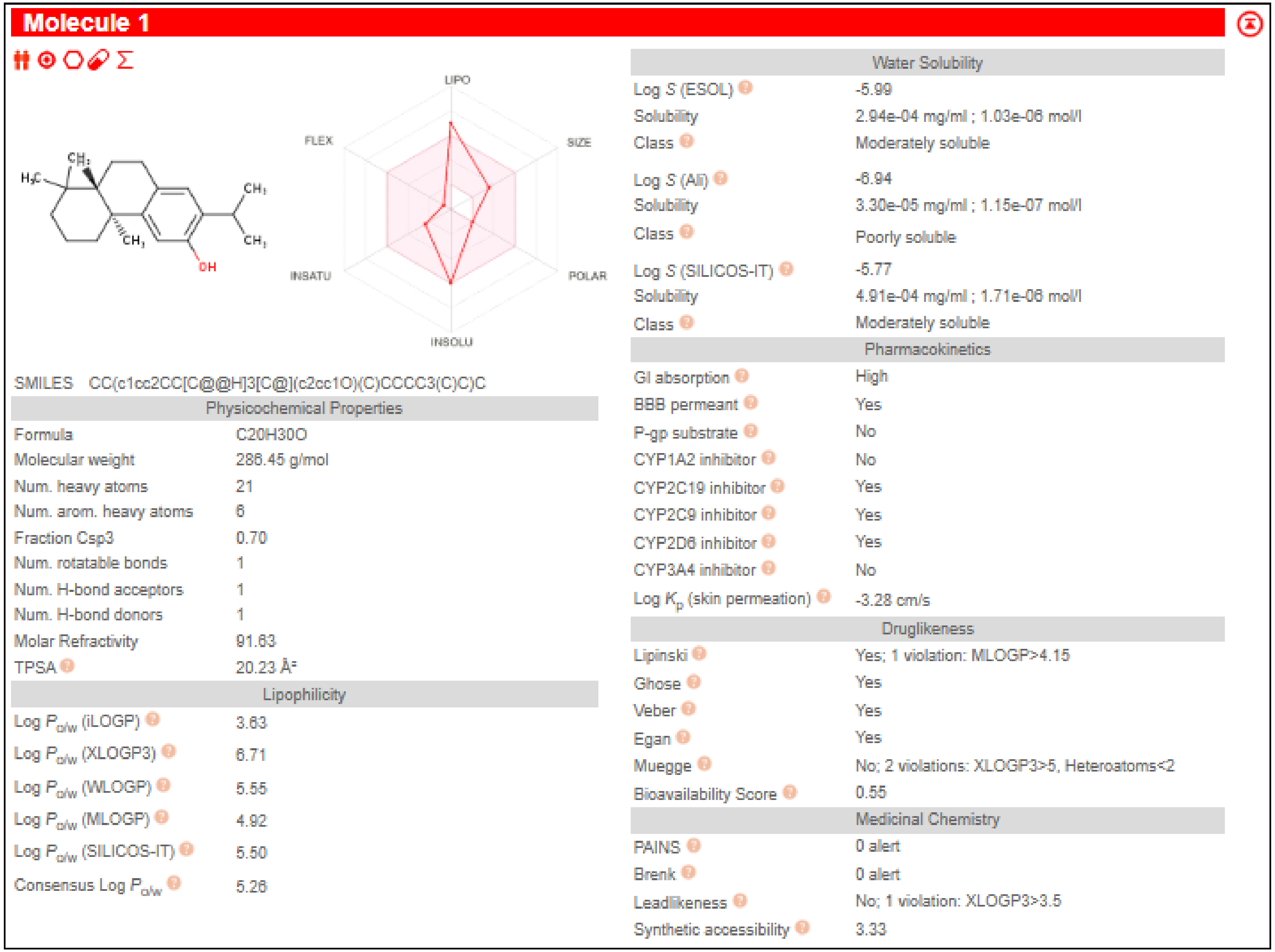

SMILES CC(c1cc2CC[C@@H]3[C@](c2cc1O)(C)CCCC3(C)C)C

Physicochemical Properties	
Formula	C20H30O
Molecular weight	286.45 g/mol
Num. heavy atoms	21
Num. arom. heavy atoms	6
Fraction Csp3	0.70
Num. rotatable bonds	1
Num. H-bond acceptors	1
Num. H-bond donors	1
Molar Refractivity	91.63
TPSA	20.23 Å²

Lipophilicity	
Log $P_{o/w}$ (iLOGP)	3.63
Log $P_{o/w}$ (XLOGP3)	6.71
Log $P_{o/w}$ (WLOGP)	5.55
Log $P_{o/w}$ (MLOGP)	4.92
Log $P_{o/w}$ (SILICOS-IT)	5.50
Consensus Log $P_{o/w}$	5.26

Water Solubility	
Log *S* (ESOL)	-5.99
Solubility	2.94e-04 mg/ml ; 1.03e-06 mol/l
Class	Moderately soluble
Log *S* (Ali)	-6.94
Solubility	3.30e-05 mg/ml ; 1.15e-07 mol/l
Class	Poorly soluble
Log *S* (SILICOS-IT)	-5.77
Solubility	4.91e-04 mg/ml ; 1.71e-06 mol/l
Class	Moderately soluble

Pharmacokinetics	
GI absorption	High
BBB permeant	Yes
P-gp substrate	No
CYP1A2 inhibitor	No
CYP2C19 inhibitor	Yes
CYP2C9 inhibitor	Yes
CYP2D6 inhibitor	Yes
CYP3A4 inhibitor	No
Log K_p (skin permeation)	-3.28 cm/s

Druglikeness	
Lipinski	Yes; 1 violation: MLOGP>4.15
Ghose	Yes
Veber	Yes
Egan	Yes
Muegge	No; 2 violations: XLOGP3>5, Heteroatoms<2
Bioavailability Score	0.55

Medicinal Chemistry	
PAINS	0 alert
Brenk	0 alert
Leadlikeness	No; 1 violation: XLOGP3>3.5
Synthetic accessibility	3.33

Figure S13. ADME-Tox and physicochemical properties of **1** predicted using the SwissADME web server (www.swissadme.ch).

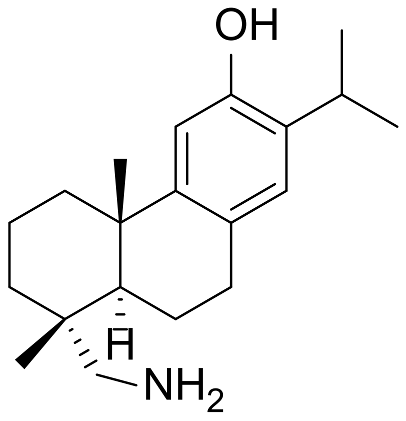

2

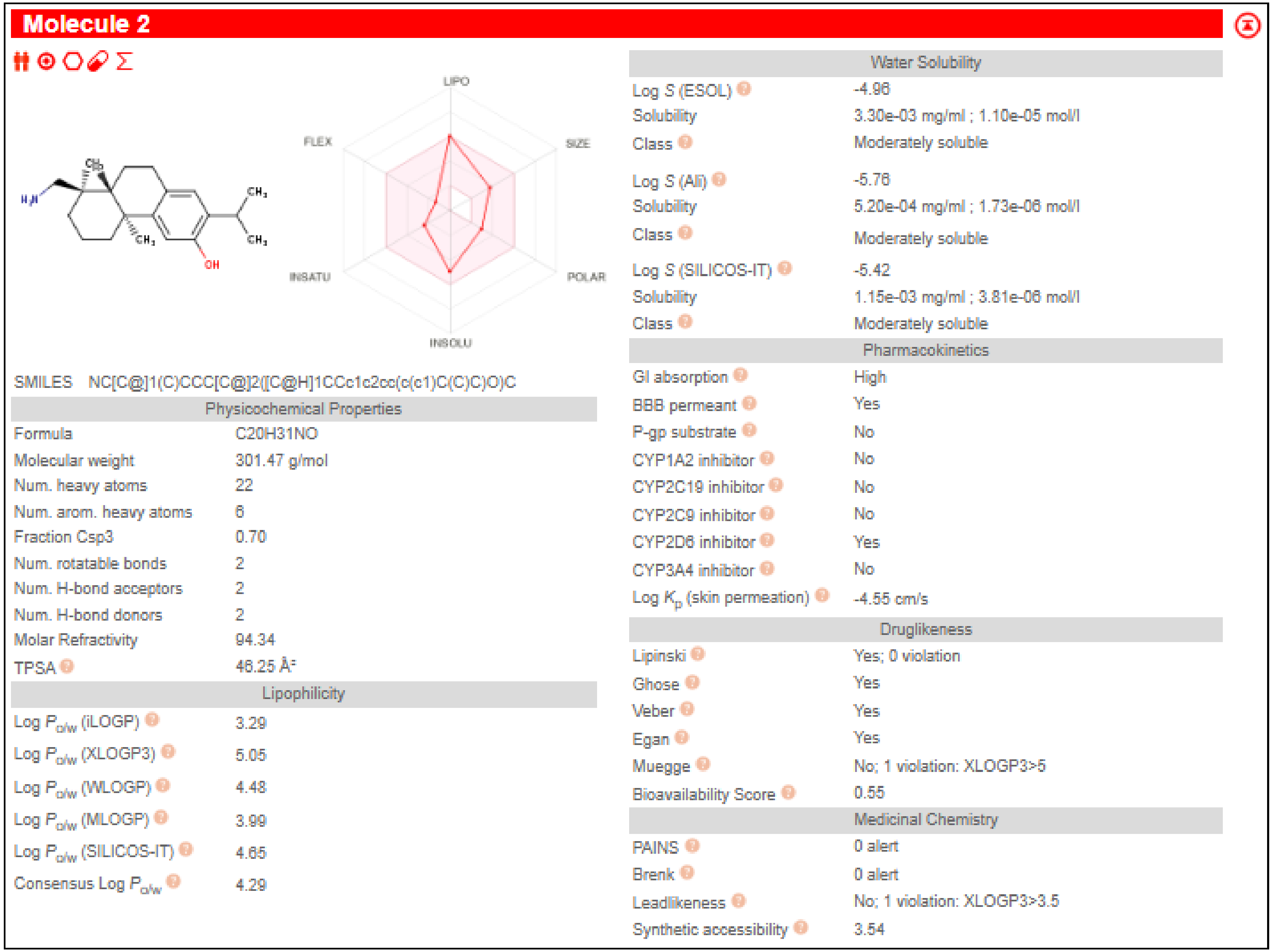

SMILES NC[C@]1(C)CCC[C@]2([C@H]1CCc1c2cc(c(c1)C(C)C)O)C

Physicochemical Properties	
Formula	C20H31NO
Molecular weight	301.47 g/mol
Num. heavy atoms	22
Num. arom. heavy atoms	6
Fraction Csp3	0.70
Num. rotatable bonds	2
Num. H-bond acceptors	2
Num. H-bond donors	2
Molar Refractivity	94.34
TPSA	46.25 Å²

Lipophilicity	
Log $P_{o/w}$ (iLOGP)	3.29
Log $P_{o/w}$ (XLOGP3)	5.05
Log $P_{o/w}$ (WLOGP)	4.48
Log $P_{o/w}$ (MLOGP)	3.99
Log $P_{o/w}$ (SILICOS-IT)	4.65
Consensus Log $P_{o/w}$	4.29

Water Solubility	
Log *S* (ESOL)	-4.96
Solubility	3.30e-03 mg/ml ; 1.10e-05 mol/l
Class	Moderately soluble
Log *S* (Ali)	-5.76
Solubility	5.20e-04 mg/ml ; 1.73e-06 mol/l
Class	Moderately soluble
Log *S* (SILICOS-IT)	-5.42
Solubility	1.15e-03 mg/ml ; 3.81e-06 mol/l
Class	Moderately soluble

Pharmacokinetics	
GI absorption	High
BBB permeant	Yes
P-gp substrate	No
CYP1A2 inhibitor	No
CYP2C19 inhibitor	No
CYP2C9 inhibitor	No
CYP2D6 inhibitor	Yes
CYP3A4 inhibitor	No
Log K_p (skin permeation)	-4.55 cm/s

Druglikeness	
Lipinski	Yes; 0 violation
Ghose	Yes
Veber	Yes
Egan	Yes
Muegge	No; 1 violation: XLOGP3>5
Bioavailability Score	0.55

Medicinal Chemistry	
PAINS	0 alert
Brenk	0 alert
Leadlikeness	No; 1 violation: XLOGP3>3.5
Synthetic accessibility	3.54

Figure S14. ADME-Tox and physicochemical properties of **2** predicted using the SwissADME web server (www.swissadme.ch).

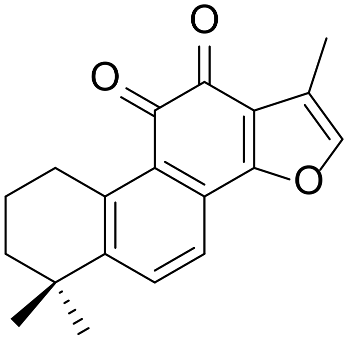

3

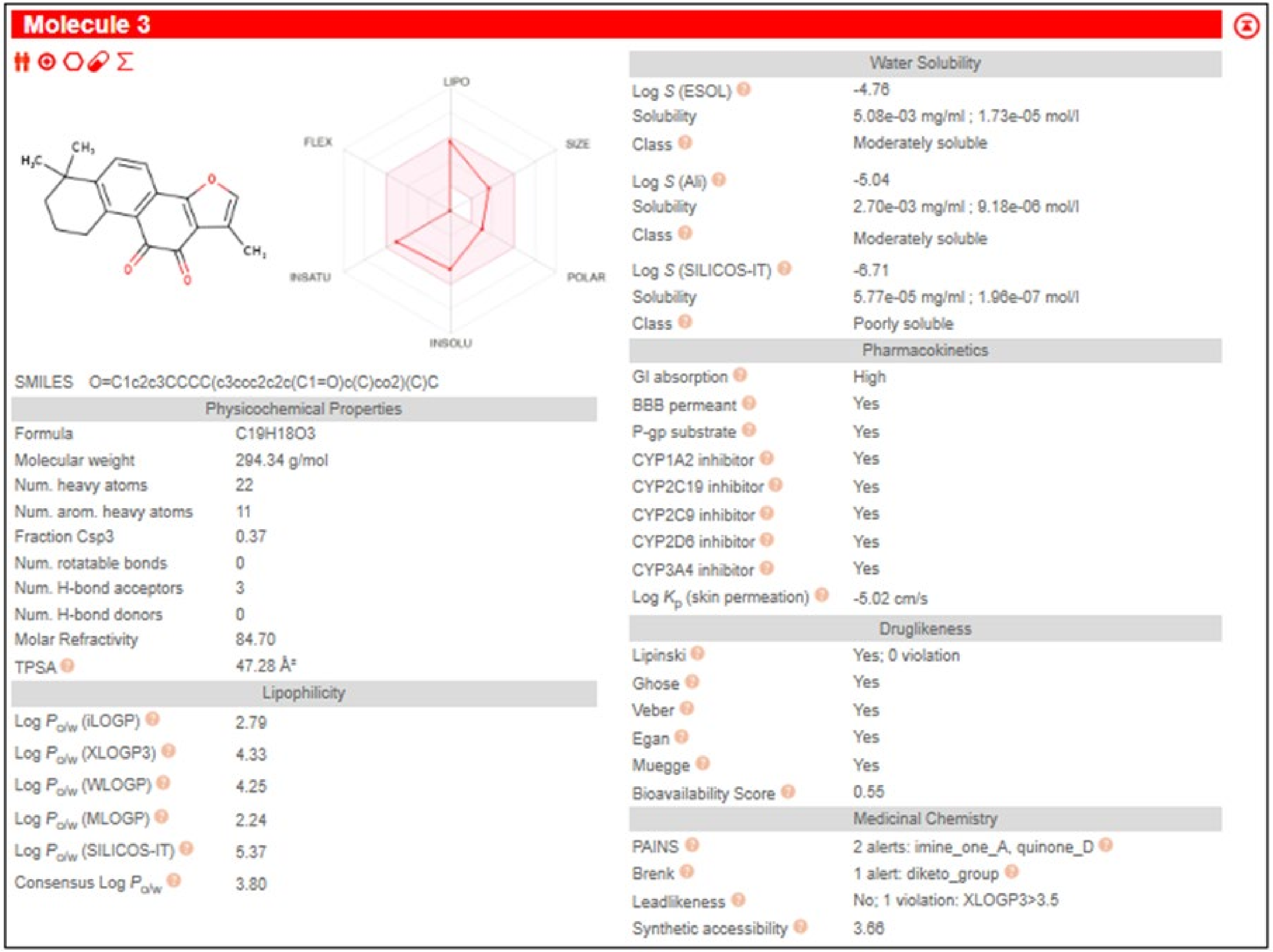

Molecule 3

SMILES O=C1c2c3CCCC(c3ccc2c2c(C1=O)c(C)co2)(C)C

Physicochemical Properties	
Formula	C19H18O3
Molecular weight	294.34 g/mol
Num. heavy atoms	22
Num. arom. heavy atoms	11
Fraction Csp3	0.37
Num. rotatable bonds	0
Num. H-bond acceptors	3
Num. H-bond donors	0
Molar Refractivity	84.70
TPSA	47.28 Å²

Lipophilicity	
Log $P_{o/w}$ (iLOGP)	2.79
Log $P_{o/w}$ (XLOGP3)	4.33
Log $P_{o/w}$ (WLOGP)	4.25
Log $P_{o/w}$ (MLOGP)	2.24
Log $P_{o/w}$ (SILICOS-IT)	5.37
Consensus Log $P_{o/w}$	3.80

Water Solubility	
Log *S* (ESOL)	-4.76
Solubility	5.08e-03 mg/ml ; 1.73e-05 mol/l
Class	Moderately soluble
Log *S* (Ali)	-5.04
Solubility	2.70e-03 mg/ml ; 9.18e-06 mol/l
Class	Moderately soluble
Log *S* (SILICOS-IT)	-6.71
Solubility	5.77e-05 mg/ml ; 1.96e-07 mol/l
Class	Poorly soluble

Pharmacokinetics	
GI absorption	High
BBB permeant	Yes
P-gp substrate	Yes
CYP1A2 inhibitor	Yes
CYP2C19 inhibitor	Yes
CYP2C9 inhibitor	Yes
CYP2D6 inhibitor	Yes
CYP3A4 inhibitor	Yes
Log K_p (skin permeation)	-5.02 cm/s

Druglikeness	
Lipinski	Yes; 0 violation
Ghose	Yes
Veber	Yes
Egan	Yes
Muegge	Yes
Bioavailability Score	0.55

Medicinal Chemistry	
PAINS	2 alerts: imine_one_A, quinone_D
Brenk	1 alert: diketo_group
Leadlikeness	No; 1 violation: XLOGP3>3.5
Synthetic accessibility	3.66

Figure S15. ADME-Tox and physicochemical properties of **3** predicted using the SwissADME web server (www.swissadme.ch).

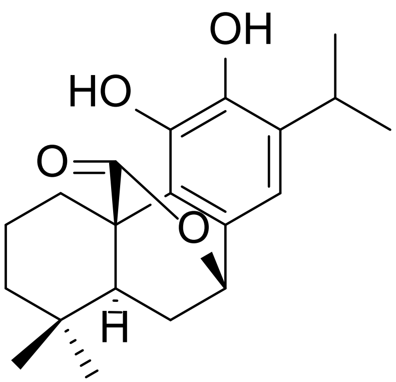

4

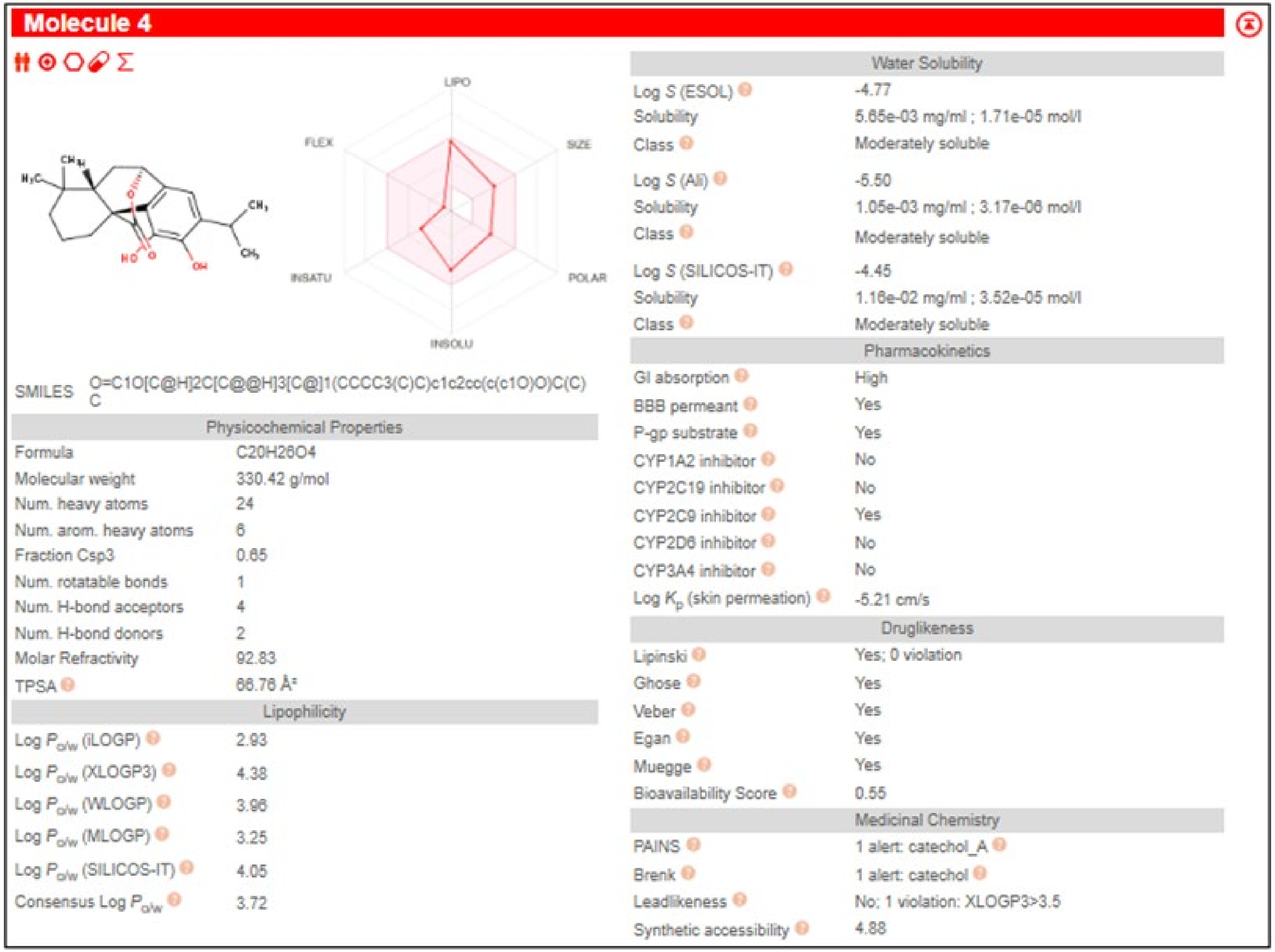

Molecule 4

SMILES: O=C1O[C@H]2C[C@@H]3[C@]1(CCCC3(C)C)c1c2cc(c(c1O)O)C(C)C

Physicochemical Properties	
Formula	C20H26O4
Molecular weight	330.42 g/mol
Num. heavy atoms	24
Num. arom. heavy atoms	6
Fraction Csp3	0.65
Num. rotatable bonds	1
Num. H-bond acceptors	4
Num. H-bond donors	2
Molar Refractivity	92.83
TPSA	66.76 Å²

Lipophilicity	
Log $P_{o/w}$ (iLOGP)	2.93
Log $P_{o/w}$ (XLOGP3)	4.38
Log $P_{o/w}$ (WLOGP)	3.96
Log $P_{o/w}$ (MLOGP)	3.25
Log $P_{o/w}$ (SILICOS-IT)	4.05
Consensus Log $P_{o/w}$	3.72

Water Solubility	
Log *S* (ESOL)	-4.77
Solubility	5.65e-03 mg/ml ; 1.71e-05 mol/l
Class	Moderately soluble
Log *S* (Ali)	-5.50
Solubility	1.05e-03 mg/ml ; 3.17e-06 mol/l
Class	Moderately soluble
Log *S* (SILICOS-IT)	-4.45
Solubility	1.16e-02 mg/ml ; 3.52e-05 mol/l
Class	Moderately soluble

Pharmacokinetics	
GI absorption	High
BBB permeant	Yes
P-gp substrate	Yes
CYP1A2 inhibitor	No
CYP2C19 inhibitor	No
CYP2C9 inhibitor	Yes
CYP2D6 inhibitor	No
CYP3A4 inhibitor	No
Log K_p (skin permeation)	-5.21 cm/s

Druglikeness	
Lipinski	Yes; 0 violation
Ghose	Yes
Veber	Yes
Egan	Yes
Muegge	Yes
Bioavailability Score	0.55

Medicinal Chemistry	
PAINS	1 alert: catechol_A
Brenk	1 alert: catechol
Leadlikeness	No; 1 violation: XLOGP3>3.5
Synthetic accessibility	4.88

Figure S16. ADME-Tox and physicochemical properties of **4** predicted using the SwissADME web server (www.swissadme.ch).

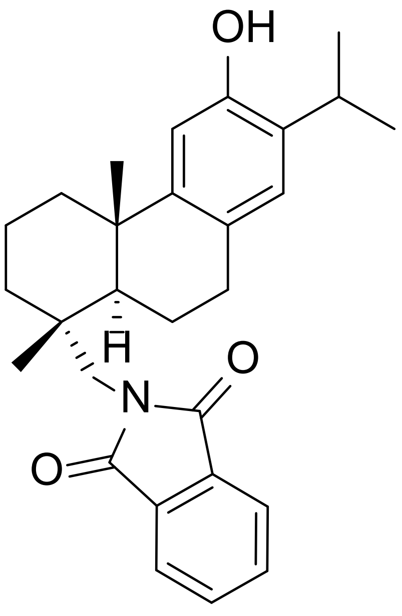

5

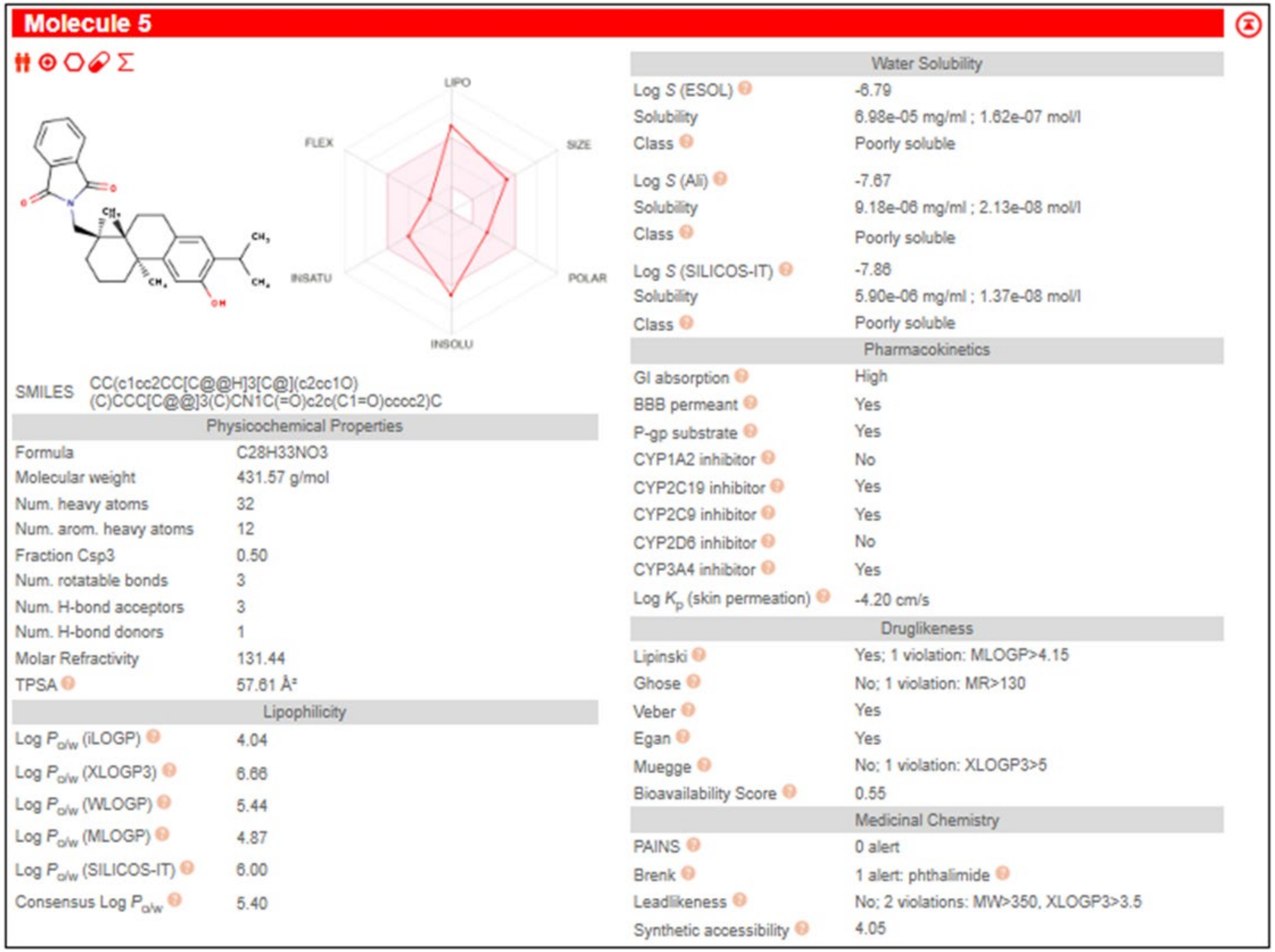

Molecule 5

SMILES: CC(c1cc2CC[C@@H]3[C@](c2cc1O)(C)CCC[C@@]3(C)CN1C(=O)c2c(C1=O)cccc2)C

Physicochemical Properties	
Formula	C28H33NO3
Molecular weight	431.57 g/mol
Num. heavy atoms	32
Num. arom. heavy atoms	12
Fraction Csp3	0.50
Num. rotatable bonds	3
Num. H-bond acceptors	3
Num. H-bond donors	1
Molar Refractivity	131.44
TPSA	57.61 Å²

Lipophilicity	
Log $P_{o/w}$ (iLOGP)	4.04
Log $P_{o/w}$ (XLOGP3)	6.66
Log $P_{o/w}$ (WLOGP)	5.44
Log $P_{o/w}$ (MLOGP)	4.87
Log $P_{o/w}$ (SILICOS-IT)	6.00
Consensus Log $P_{o/w}$	5.40

Water Solubility	
Log *S* (ESOL)	-6.79
Solubility	6.98e-05 mg/ml ; 1.62e-07 mol/l
Class	Poorly soluble
Log *S* (Ali)	-7.67
Solubility	9.18e-06 mg/ml ; 2.13e-08 mol/l
Class	Poorly soluble
Log *S* (SILICOS-IT)	-7.86
Solubility	5.90e-06 mg/ml ; 1.37e-08 mol/l
Class	Poorly soluble

Pharmacokinetics	
GI absorption	High
BBB permeant	Yes
P-gp substrate	Yes
CYP1A2 inhibitor	No
CYP2C19 inhibitor	Yes
CYP2C9 inhibitor	Yes
CYP2D6 inhibitor	No
CYP3A4 inhibitor	Yes
Log K_p (skin permeation)	-4.20 cm/s

Druglikeness	
Lipinski	Yes; 1 violation: MLOGP>4.15
Ghose	No; 1 violation: MR>130
Veber	Yes
Egan	Yes
Muegge	No; 1 violation: XLOGP3>5
Bioavailability Score	0.55

Medicinal Chemistry	
PAINS	0 alert
Brenk	1 alert: phthalimide
Leadlikeness	No; 2 violations: MW>350, XLOGP3>3.5
Synthetic accessibility	4.05

Figure S17. ADME-Tox and physicochemical properties of **5** predicted using the SwissADME web server (www.swissadme.ch).

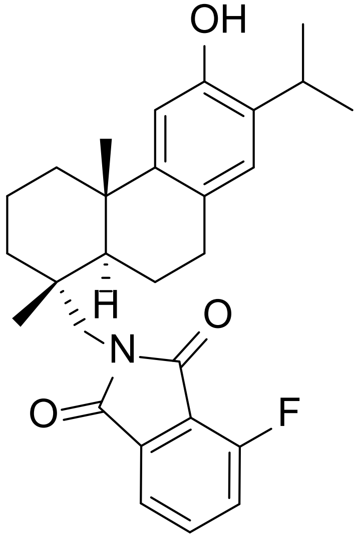

6

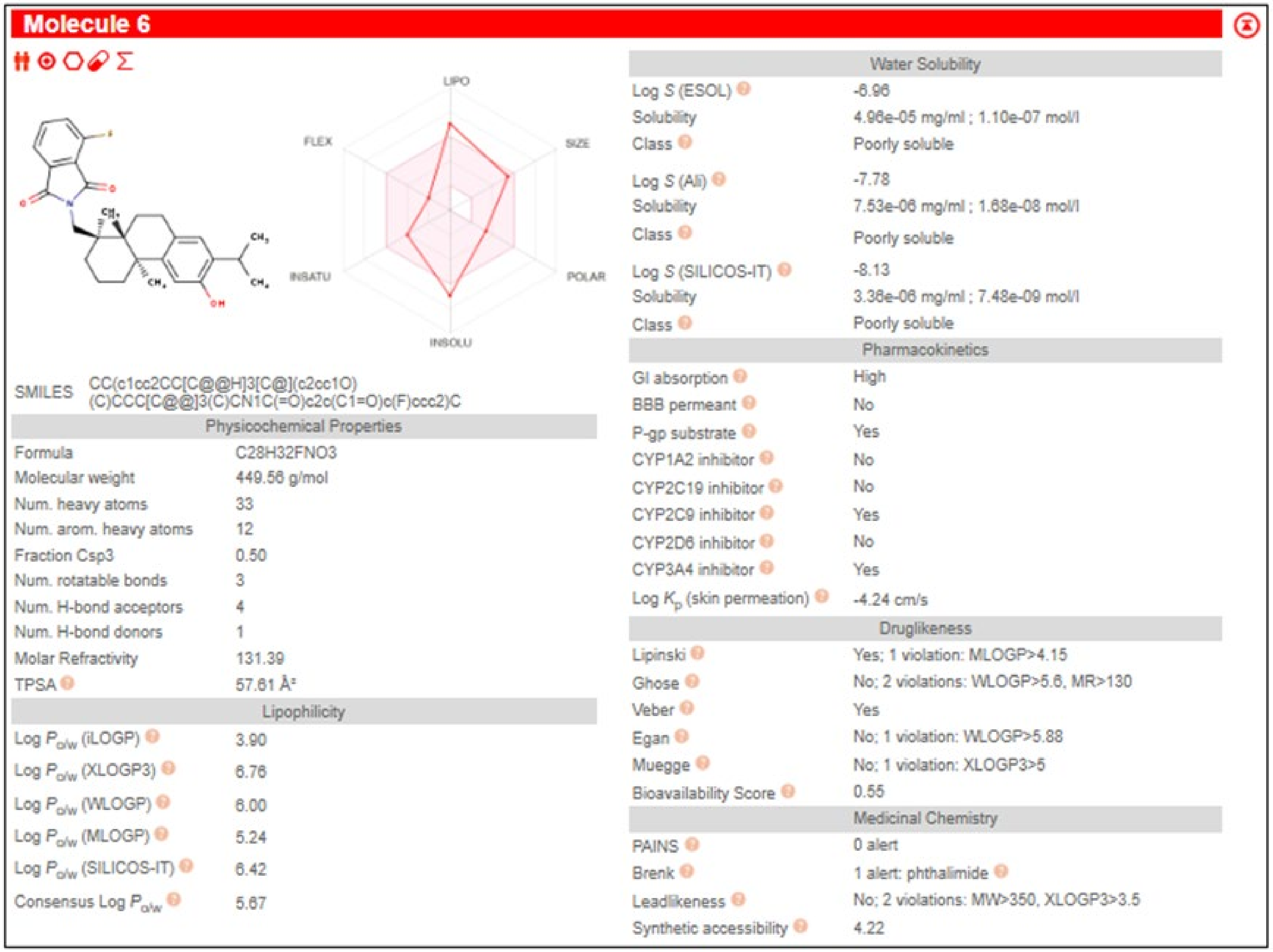
Molecule 6

SMILES CC(c1cc2CC[C@@H]3[C@](c2cc1O)(C)CCC[C@@]3(C)CN1C(=O)c2c(C1=O)c(F)ccc2)C

Physicochemical Properties	
Formula	C28H32FNO3
Molecular weight	449.56 g/mol
Num. heavy atoms	33
Num. arom. heavy atoms	12
Fraction Csp3	0.50
Num. rotatable bonds	3
Num. H-bond acceptors	4
Num. H-bond donors	1
Molar Refractivity	131.39
TPSA	57.61 Å²

Lipophilicity	
Log $P_{o/w}$ (iLOGP)	3.90
Log $P_{o/w}$ (XLOGP3)	6.76
Log $P_{o/w}$ (WLOGP)	6.00
Log $P_{o/w}$ (MLOGP)	5.24
Log $P_{o/w}$ (SILICOS-IT)	6.42
Consensus Log $P_{o/w}$	5.67

Water Solubility	
Log *S* (ESOL)	-6.96
Solubility	4.96e-05 mg/ml ; 1.10e-07 mol/l
Class	Poorly soluble
Log *S* (Ali)	-7.78
Solubility	7.53e-06 mg/ml ; 1.68e-08 mol/l
Class	Poorly soluble
Log *S* (SILICOS-IT)	-8.13
Solubility	3.36e-06 mg/ml ; 7.48e-09 mol/l
Class	Poorly soluble

Pharmacokinetics	
GI absorption	High
BBB permeant	No
P-gp substrate	Yes
CYP1A2 inhibitor	No
CYP2C19 inhibitor	No
CYP2C9 inhibitor	Yes
CYP2D6 inhibitor	No
CYP3A4 inhibitor	Yes
Log K_p (skin permeation)	-4.24 cm/s

Druglikeness	
Lipinski	Yes; 1 violation: MLOGP>4.15
Ghose	No; 2 violations: WLOGP>5.6, MR>130
Veber	Yes
Egan	No; 1 violation: WLOGP>5.88
Muegge	No; 1 violation: XLOGP3>5
Bioavailability Score	0.55

Medicinal Chemistry	
PAINS	0 alert
Brenk	1 alert: phthalimide
Leadlikeness	No; 2 violations: MW>350, XLOGP3>3.5
Synthetic accessibility	4.22

Figure S18. ADME-Tox and physicochemical properties of **6** predicted using the SwissADME web server (www.swissadme.ch).

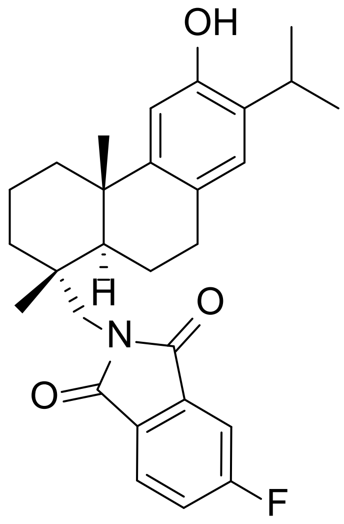

7

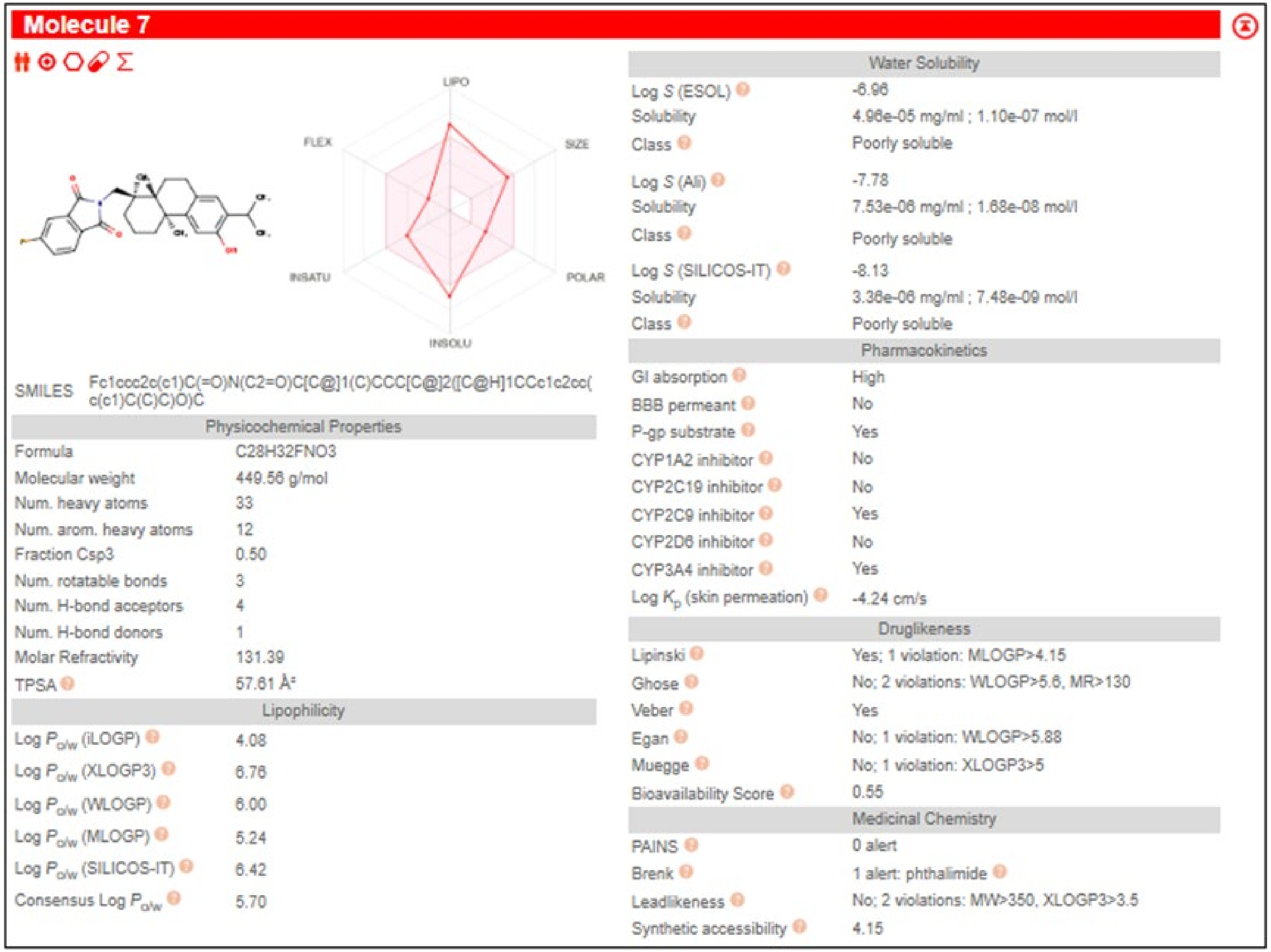

SMILES Fc1ccc2c(c1)C(=O)N(C2=O)C[C@]1(C)CCC[C@]2([C@H]1CCc1c2cc(c(c1)C(C)C)O)C

Physicochemical Properties	
Formula	C28H32FNO3
Molecular weight	449.56 g/mol
Num. heavy atoms	33
Num. arom. heavy atoms	12
Fraction Csp3	0.50
Num. rotatable bonds	3
Num. H-bond acceptors	4
Num. H-bond donors	1
Molar Refractivity	131.39
TPSA	57.61 Å²

Lipophilicity	
Log $P_{o/w}$ (iLOGP)	4.08
Log $P_{o/w}$ (XLOGP3)	6.76
Log $P_{o/w}$ (WLOGP)	6.00
Log $P_{o/w}$ (MLOGP)	5.24
Log $P_{o/w}$ (SILICOS-IT)	6.42
Consensus Log $P_{o/w}$	5.70

Water Solubility	
Log *S* (ESOL)	-6.96
Solubility	4.96e-05 mg/ml ; 1.10e-07 mol/l
Class	Poorly soluble
Log *S* (Ali)	-7.78
Solubility	7.53e-06 mg/ml ; 1.68e-08 mol/l
Class	Poorly soluble
Log *S* (SILICOS-IT)	-8.13
Solubility	3.36e-06 mg/ml ; 7.48e-09 mol/l
Class	Poorly soluble

Pharmacokinetics	
GI absorption	High
BBB permeant	No
P-gp substrate	Yes
CYP1A2 inhibitor	No
CYP2C19 inhibitor	No
CYP2C9 inhibitor	Yes
CYP2D6 inhibitor	No
CYP3A4 inhibitor	Yes
Log K_p (skin permeation)	-4.24 cm/s

Druglikeness	
Lipinski	Yes; 1 violation: MLOGP>4.15
Ghose	No; 2 violations: WLOGP>5.6, MR>130
Veber	Yes
Egan	No; 1 violation: WLOGP>5.88
Muegge	No; 1 violation: XLOGP3>5
Bioavailability Score	0.55

Medicinal Chemistry	
PAINS	0 alert
Brenk	1 alert: phthalimide
Leadlikeness	No; 2 violations: MW>350, XLOGP3>3.5
Synthetic accessibility	4.15

Figure S19. ADME-Tox and physicochemical properties of **7** predicted using the SwissADME web server (www.swissadme.ch).

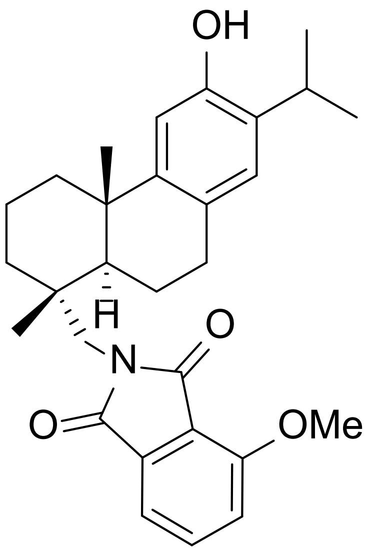

8

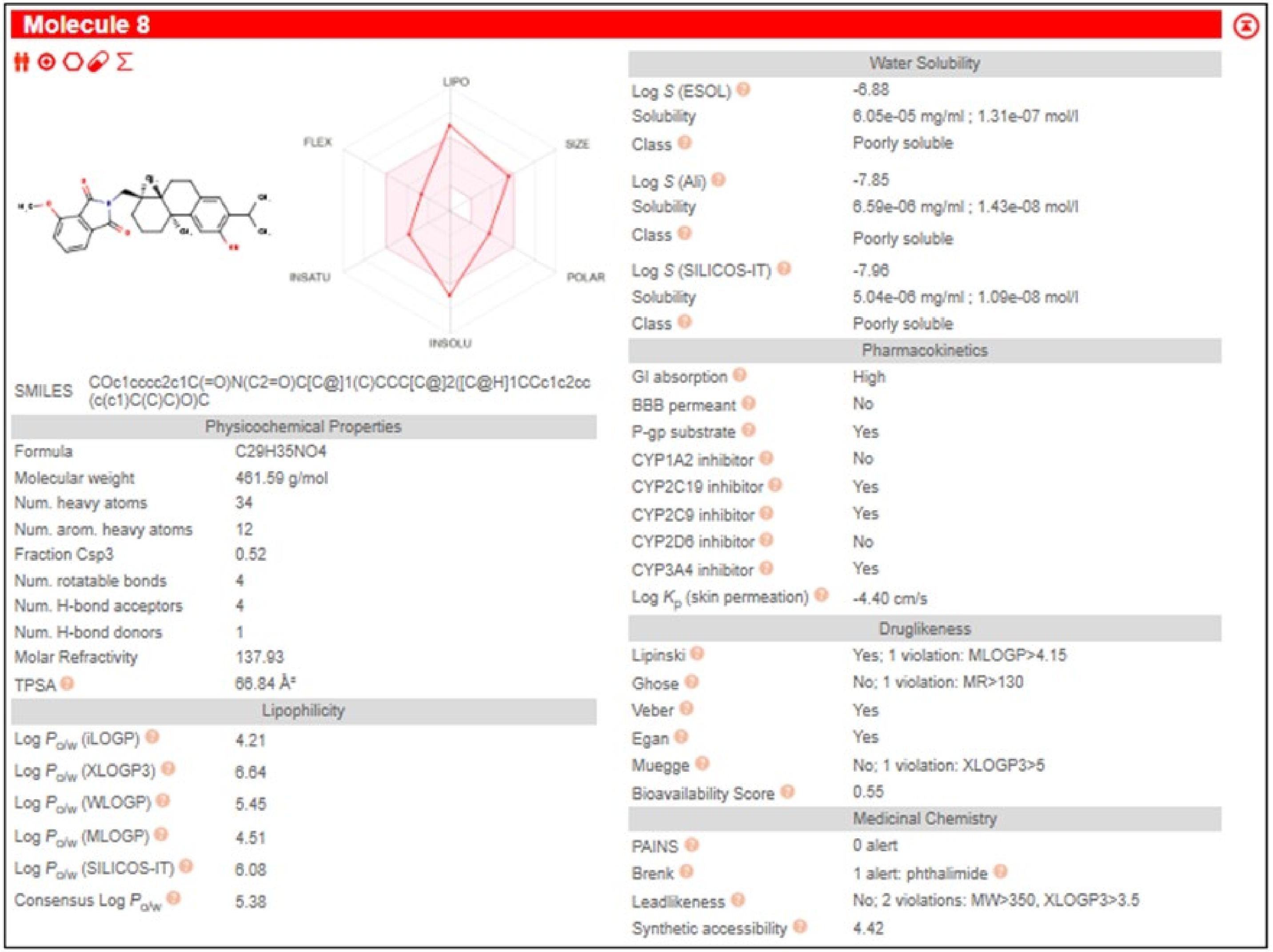

SMILES COc1cccc2c1C(=O)N(C2=O)C[C@]1(C)CCC[C@]2([C@H]1CCc1c2cc(c(c1)C(C)C)O)C

Physicochemical Properties	
Formula	C29H35NO4
Molecular weight	461.59 g/mol
Num. heavy atoms	34
Num. arom. heavy atoms	12
Fraction Csp3	0.52
Num. rotatable bonds	4
Num. H-bond acceptors	4
Num. H-bond donors	1
Molar Refractivity	137.93
TPSA	66.84 Å²

Lipophilicity	
Log $P_{o/w}$ (iLOGP)	4.21
Log $P_{o/w}$ (XLOGP3)	6.64
Log $P_{o/w}$ (WLOGP)	5.45
Log $P_{o/w}$ (MLOGP)	4.51
Log $P_{o/w}$ (SILICOS-IT)	6.08
Consensus Log $P_{o/w}$	5.38

Water Solubility	
Log *S* (ESOL)	-6.88
Solubility	6.05e-05 mg/ml ; 1.31e-07 mol/l
Class	Poorly soluble
Log *S* (Ali)	-7.85
Solubility	6.59e-06 mg/ml ; 1.43e-08 mol/l
Class	Poorly soluble
Log *S* (SILICOS-IT)	-7.96
Solubility	5.04e-06 mg/ml ; 1.09e-08 mol/l
Class	Poorly soluble

Pharmacokinetics	
GI absorption	High
BBB permeant	No
P-gp substrate	Yes
CYP1A2 inhibitor	No
CYP2C19 inhibitor	Yes
CYP2C9 inhibitor	Yes
CYP2D6 inhibitor	No
CYP3A4 inhibitor	Yes
Log K_p (skin permeation)	-4.40 cm/s

Druglikeness	
Lipinski	Yes; 1 violation: MLOGP>4.15
Ghose	No; 1 violation: MR>130
Veber	Yes
Egan	Yes
Muegge	No; 1 violation: XLOGP3>5
Bioavailability Score	0.55

Medicinal Chemistry	
PAINS	0 alert
Brenk	1 alert: phthalimide
Leadlikeness	No; 2 violations: MW>350, XLOGP3>3.5
Synthetic accessibility	4.42

Figure S20. ADME-Tox and physicochemical properties of **8** predicted using the SwissADME web server (www.swissadme.ch).

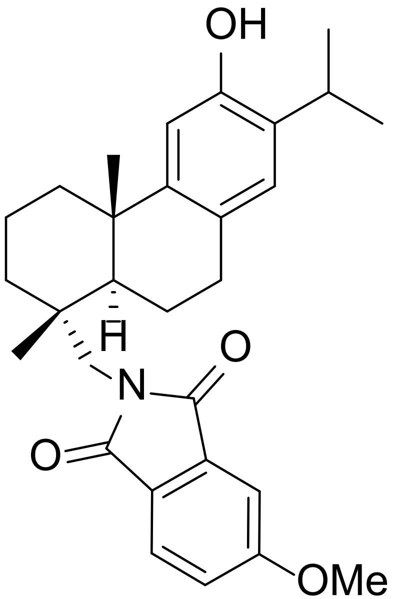

9

Molecule 9

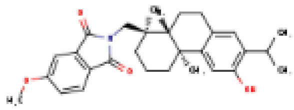

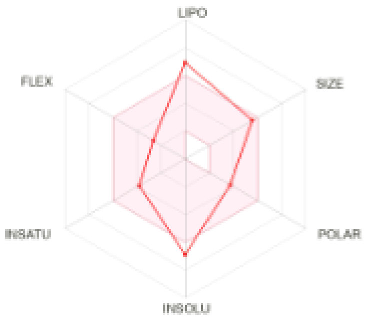

SMILES COc1ccc2c(c1)C(=O)N(C2=O)C[C@]1(C)CCC[C@]2([C@H]1CCc1c2cc(c(c1)C(C)C)O)C

Physicochemical Properties	
Formula	C29H35NO4
Molecular weight	461.59 g/mol
Num. heavy atoms	34
Num. arom. heavy atoms	12
Fraction Csp3	0.52
Num. rotatable bonds	4
Num. H-bond acceptors	4
Num. H-bond donors	1
Molar Refractivity	137.93
TPSA	66.84 Å²

Lipophilicity	
Log $P_{o/w}$ (iLOGP)	4.25
Log $P_{o/w}$ (XLOGP3)	6.64
Log $P_{o/w}$ (WLOGP)	5.45
Log $P_{o/w}$ (MLOGP)	4.51
Log $P_{o/w}$ (SILICOS-IT)	6.08
Consensus Log $P_{o/w}$	5.38

Water Solubility	
Log *S* (ESOL)	-6.88
Solubility	6.05e-05 mg/ml ; 1.31e-07 mol/l
Class	Poorly soluble
Log *S* (Ali)	-7.85
Solubility	6.59e-06 mg/ml ; 1.43e-08 mol/l
Class	Poorly soluble
Log *S* (SILICOS-IT)	-7.96
Solubility	5.04e-06 mg/ml ; 1.09e-08 mol/l
Class	Poorly soluble

Pharmacokinetics	
GI absorption	High
BBB permeant	No
P-gp substrate	Yes
CYP1A2 inhibitor	No
CYP2C19 inhibitor	Yes
CYP2C9 inhibitor	Yes
CYP2D6 inhibitor	No
CYP3A4 inhibitor	Yes
Log K_p (skin permeation)	-4.40 cm/s

Druglikeness	
Lipinski	Yes; 1 violation: MLOGP>4.15
Ghose	No; 1 violation: MR>130
Veber	Yes
Egan	Yes
Muegge	No; 1 violation: XLOGP3>5
Bioavailability Score	0.55

Medicinal Chemistry	
PAINS	0 alert
Brenk	1 alert: phthalimide
Leadlikeness	No; 2 violations: MW>350, XLOGP3>3.5
Synthetic accessibility	4.34

Figure S21. ADME-Tox and physicochemical properties of **9** predicted using the SwissADME web server (www.swissadme.ch).

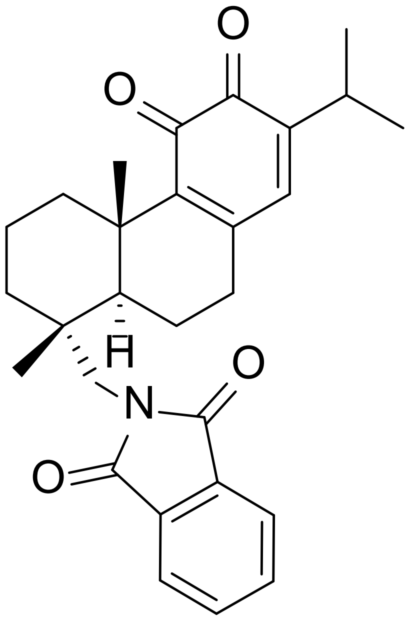

10

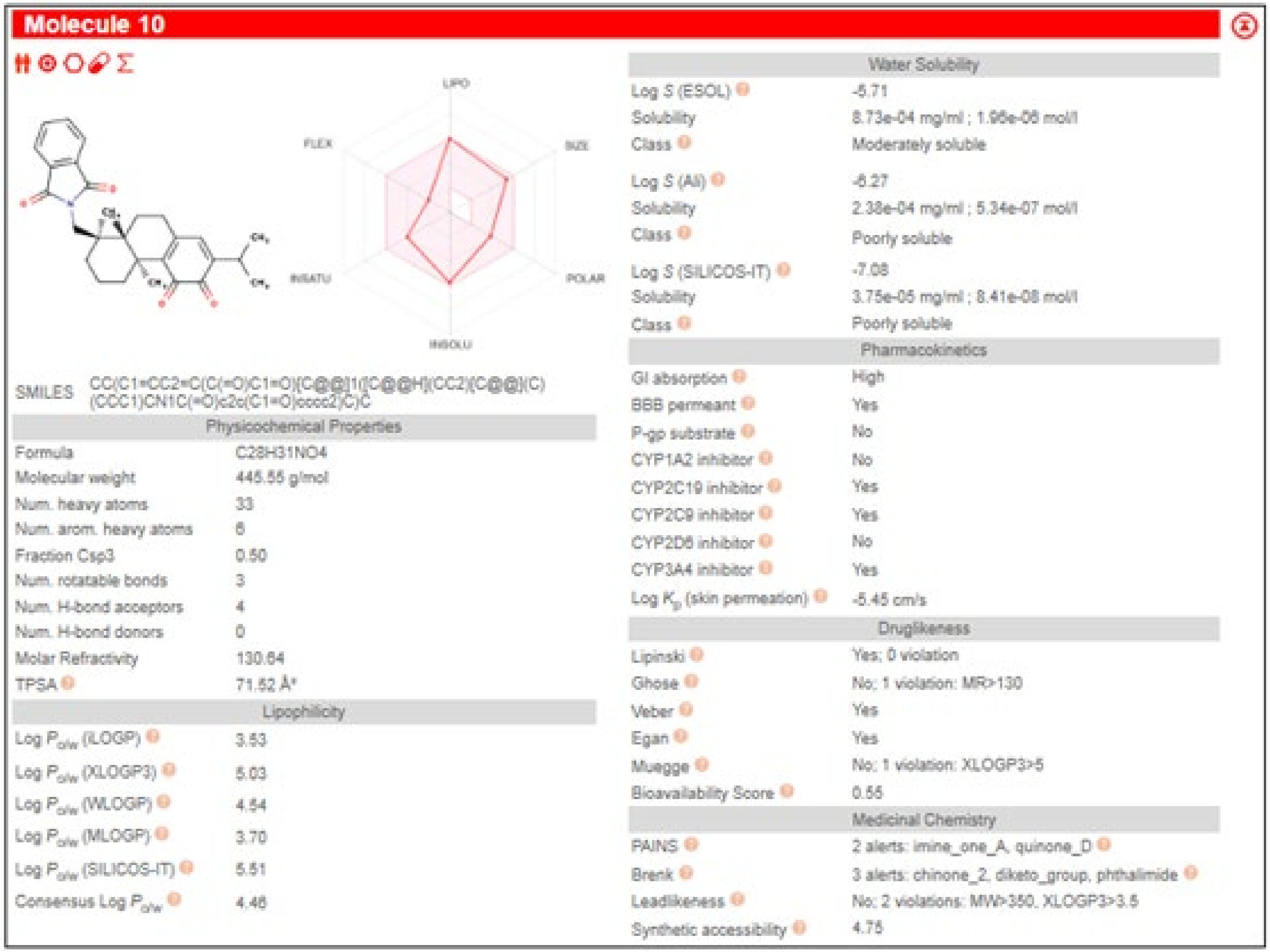

Molecule 10

SMILES CC(C1=CC2=C(C(=O)C1=O)[C@@]1([C@@H](CC2)[C@@](C)(CCC1)CN1C(=O)c2c(C1=O)cccc2)C)C

Physicochemical Properties	
Formula	C28H31NO4
Molecular weight	445.55 g/mol
Num. heavy atoms	33
Num. arom. heavy atoms	6
Fraction Csp3	0.50
Num. rotatable bonds	3
Num. H-bond acceptors	4
Num. H-bond donors	0
Molar Refractivity	130.64
TPSA	71.52 Å²

Lipophilicity	
Log $P_{o/w}$ (iLOGP)	3.53
Log $P_{o/w}$ (XLOGP3)	5.03
Log $P_{o/w}$ (WLOGP)	4.54
Log $P_{o/w}$ (MLOGP)	3.70
Log $P_{o/w}$ (SILICOS-IT)	5.51
Consensus Log $P_{o/w}$	4.46

Water Solubility	
Log *S* (ESOL)	-5.71
Solubility	8.73e-04 mg/ml ; 1.96e-06 mol/l
Class	Moderately soluble
Log *S* (Ali)	-6.27
Solubility	2.38e-04 mg/ml ; 5.34e-07 mol/l
Class	Poorly soluble
Log *S* (SILICOS-IT)	-7.08
Solubility	3.75e-05 mg/ml ; 8.41e-08 mol/l
Class	Poorly soluble

Pharmacokinetics	
GI absorption	High
BBB permeant	Yes
P-gp substrate	No
CYP1A2 inhibitor	No
CYP2C19 inhibitor	Yes
CYP2C9 inhibitor	Yes
CYP2D6 inhibitor	No
CYP3A4 inhibitor	Yes
Log K_p (skin permeation)	-5.45 cm/s

Druglikeness	
Lipinski	Yes; 0 violation
Ghose	No; 1 violation: MR>130
Veber	Yes
Egan	Yes
Muegge	No; 1 violation: XLOGP3>5
Bioavailability Score	0.55

Medicinal Chemistry	
PAINS	2 alerts: imine_one_A, quinone_D
Brenk	3 alerts: chinone_2, diketo_group, phthalimide
Leadlikeness	No; 2 violations: MW>350, XLOGP3>3.5
Synthetic accessibility	4.75

Figure S22. ADME-Tox and physicochemical properties of **10** predicted using the SwissADME web server (www.swissadme.ch).

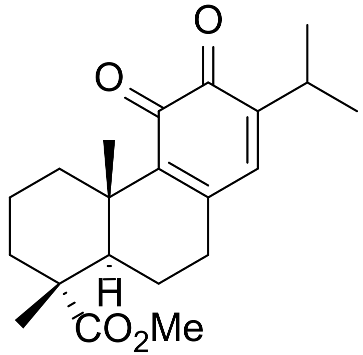

11

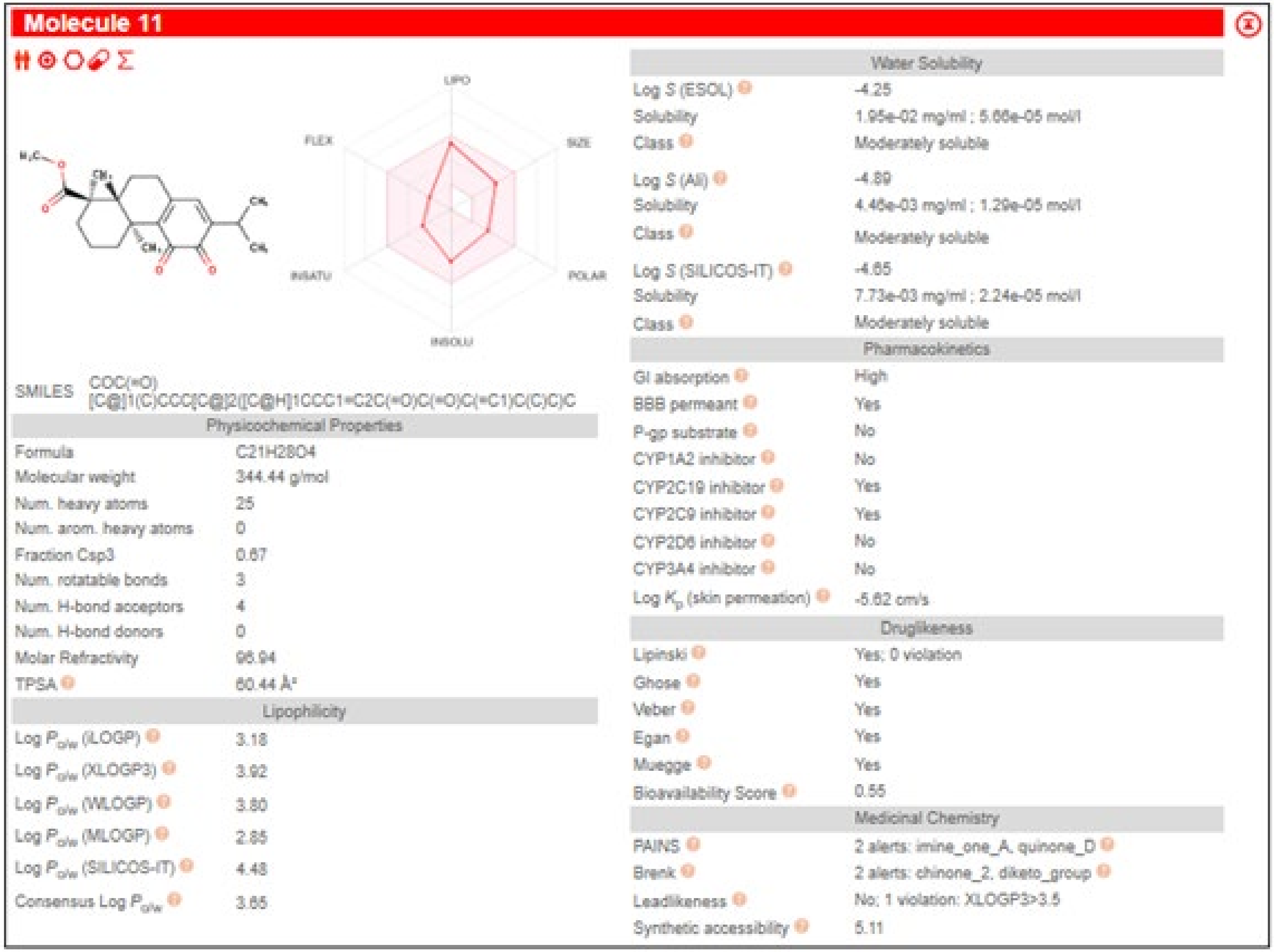

Molecule 11

SMILES COC(=O)[C@]1(C)CCC[C@]2([C@H]1CCC1=C2C(=O)C(=O)C(=C1)C(C)C)C

Physicochemical Properties	
Formula	C21H28O4
Molecular weight	344.44 g/mol
Num. heavy atoms	25
Num. arom. heavy atoms	0
Fraction Csp3	0.67
Num. rotatable bonds	3
Num. H-bond acceptors	4
Num. H-bond donors	0
Molar Refractivity	96.94
TPSA	60.44 Å²

Lipophilicity	
Log $P_{o/w}$ (iLOGP)	3.18
Log $P_{o/w}$ (XLOGP3)	3.92
Log $P_{o/w}$ (WLOGP)	3.80
Log $P_{o/w}$ (MLOGP)	2.85
Log $P_{o/w}$ (SILICOS-IT)	4.48
Consensus Log $P_{o/w}$	3.65

Water Solubility	
Log *S* (ESOL)	-4.25
Solubility	1.95e-02 mg/ml ; 5.66e-05 mol/l
Class	Moderately soluble
Log *S* (Ali)	-4.89
Solubility	4.45e-03 mg/ml ; 1.29e-05 mol/l
Class	Moderately soluble
Log *S* (SILICOS-IT)	-4.65
Solubility	7.73e-03 mg/ml ; 2.24e-05 mol/l
Class	Moderately soluble

Pharmacokinetics	
GI absorption	High
BBB permeant	Yes
P-gp substrate	No
CYP1A2 inhibitor	No
CYP2C19 inhibitor	Yes
CYP2C9 inhibitor	Yes
CYP2D6 inhibitor	No
CYP3A4 inhibitor	No
Log K_p (skin permeation)	-5.62 cm/s

Druglikeness	
Lipinski	Yes; 0 violation
Ghose	Yes
Veber	Yes
Egan	Yes
Muegge	Yes
Bioavailability Score	0.55

Medicinal Chemistry	
PAINS	2 alerts: imine_one_A, quinone_D
Brenk	2 alerts: chinone_2, diketo_group
Leadlikeness	No; 1 violation: XLOGP3>3.5
Synthetic accessibility	5.11

Figure S23. ADME-Tox and physicochemical properties of **11** predicted using the SwissADME web server (www.swissadme.ch).

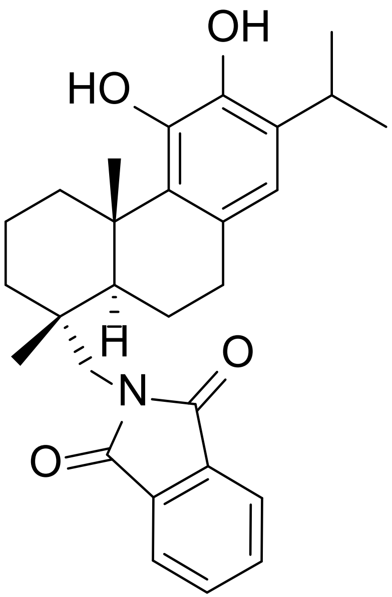

12

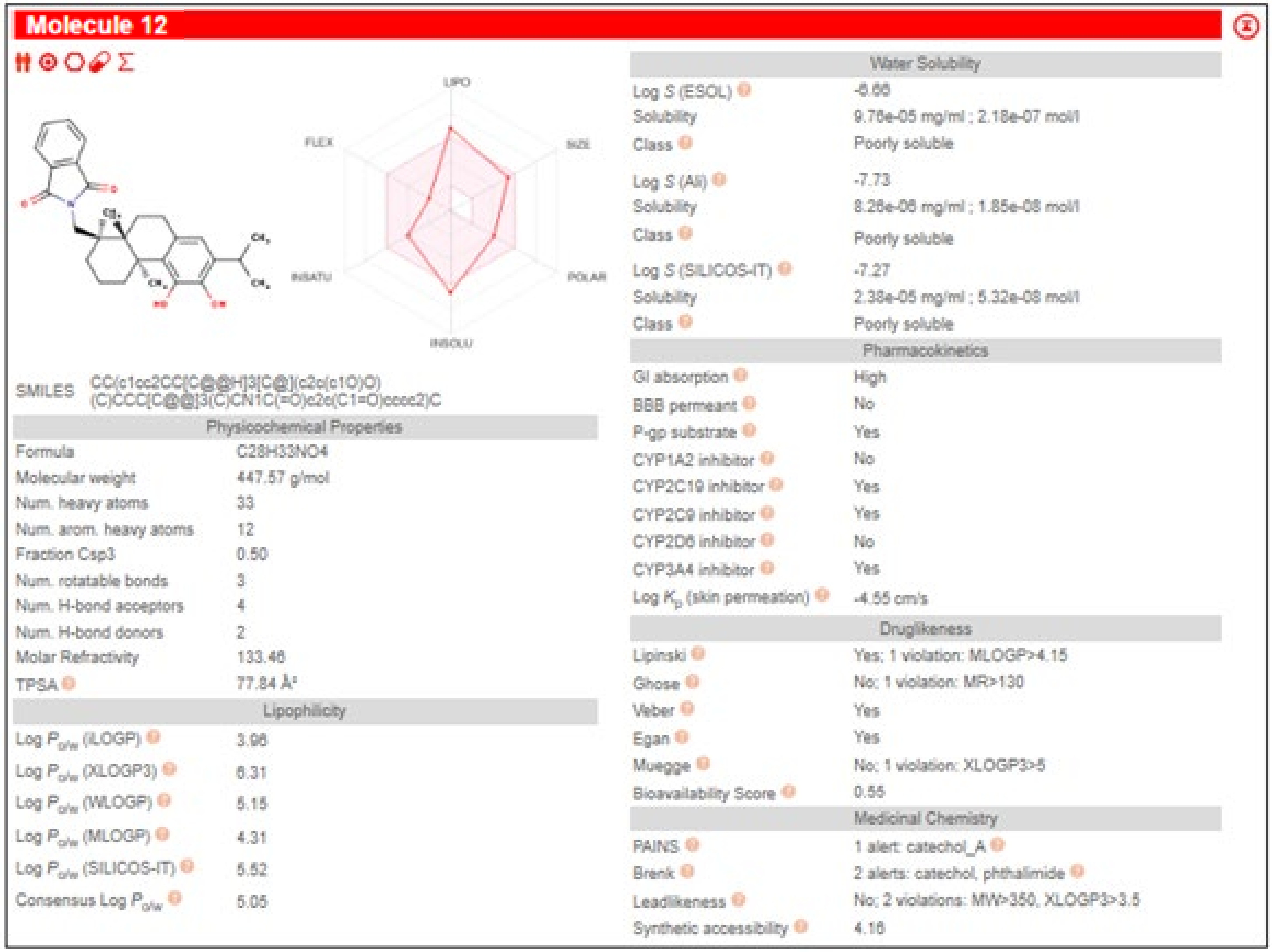

Molecule 12

SMILES CC(c1cc2CC[C@@H]3[C@](c2c(c1O)O)(C)CCC[C@@]3(C)CN1C(=O)c2c(C1=O)cccc2)C

Physicochemical Properties	
Formula	C28H33NO4
Molecular weight	447.57 g/mol
Num. heavy atoms	33
Num. arom. heavy atoms	12
Fraction Csp3	0.50
Num. rotatable bonds	3
Num. H-bond acceptors	4
Num. H-bond donors	2
Molar Refractivity	133.46
TPSA	77.84 Å²

Lipophilicity	
Log $P_{o/w}$ (iLOGP)	3.96
Log $P_{o/w}$ (XLOGP3)	6.31
Log $P_{o/w}$ (WLOGP)	5.15
Log $P_{o/w}$ (MLOGP)	4.31
Log $P_{o/w}$ (SILICOS-IT)	5.52
Consensus Log $P_{o/w}$	5.05

Water Solubility	
Log *S* (ESOL)	-6.66
Solubility	9.76e-05 mg/ml ; 2.18e-07 mol/l
Class	Poorly soluble
Log *S* (Ali)	-7.73
Solubility	8.26e-06 mg/ml ; 1.85e-08 mol/l
Class	Poorly soluble
Log *S* (SILICOS-IT)	-7.27
Solubility	2.38e-05 mg/ml ; 5.32e-08 mol/l
Class	Poorly soluble

Pharmacokinetics	
GI absorption	High
BBB permeant	No
P-gp substrate	Yes
CYP1A2 inhibitor	No
CYP2C19 inhibitor	Yes
CYP2C9 inhibitor	Yes
CYP2D6 inhibitor	No
CYP3A4 inhibitor	Yes
Log K_p (skin permeation)	-4.55 cm/s

Druglikeness	
Lipinski	Yes; 1 violation: MLOGP>4.15
Ghose	No; 1 violation: MR>130
Veber	Yes
Egan	Yes
Muegge	No; 1 violation: XLOGP3>5
Bioavailability Score	0.55

Medicinal Chemistry	
PAINS	1 alert: catechol_A
Brenk	2 alerts: catechol, phthalimide
Leadlikeness	No; 2 violations: MW>350, XLOGP3>3.5
Synthetic accessibility	4.16

Figure S24. ADME-Tox and physicochemical properties of **12** predicted using the SwissADME web server (www.swissadme.ch).

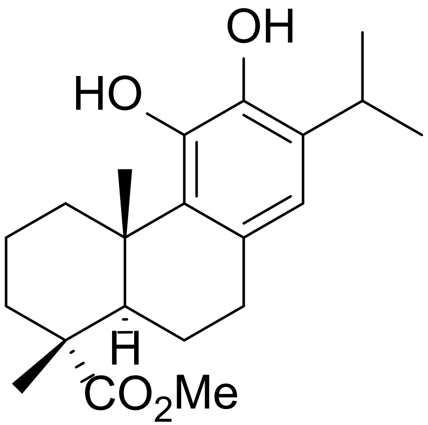

13

Molecule 13

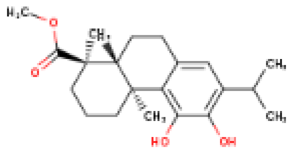

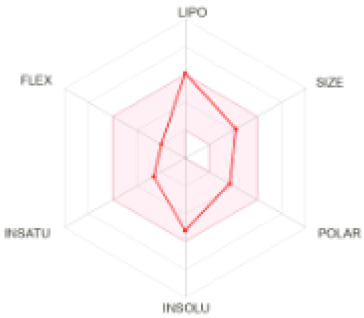

SMILES COC(=O)[C@]1(C)CCC[C@]2([C@H]1CCc1c2c(O)c(c(c1)C(C)C)O)C

Physicochemical Properties	
Formula	C21H30O4
Molecular weight	346.46 g/mol
Num. heavy atoms	25
Num. arom. heavy atoms	6
Fraction Csp3	0.67
Num. rotatable bonds	3
Num. H-bond acceptors	4
Num. H-bond donors	2
Molar Refractivity	99.75
TPSA	66.76 Å²

Lipophilicity	
Log $P_{o/w}$ (iLOGP)	3.53
Log $P_{o/w}$ (XLOGP3)	5.20
Log $P_{o/w}$ (WLOGP)	4.40
Log $P_{o/w}$ (MLOGP)	3.48
Log $P_{o/w}$ (SILICOS-IT)	4.50
Consensus Log $P_{o/w}$	4.22

Water Solubility	
Log *S* (ESOL)	-5.24
Solubility	1.98e-03 mg/ml ; 5.71e-06 mol/l
Class	Moderately soluble
Log *S* (Ali)	-6.35
Solubility	1.55e-04 mg/ml ; 4.48e-07 mol/l
Class	Poorly soluble
Log *S* (SILICOS-IT)	-4.85
Solubility	4.91e-03 mg/ml ; 1.42e-05 mol/l
Class	Moderately soluble

Pharmacokinetics	
GI absorption	High
BBB permeant	Yes
P-gp substrate	No
CYP1A2 inhibitor	No
CYP2C19 inhibitor	No
CYP2C9 inhibitor	Yes
CYP2D6 inhibitor	Yes
CYP3A4 inhibitor	No
Log K_p (skin permeation)	-4.72 cm/s

Druglikeness	
Lipinski	Yes; 0 violation
Ghose	Yes
Veber	Yes
Egan	Yes
Muegge	No; 1 violation: XLOGP3>5
Bioavailability Score	0.55

Medicinal Chemistry	
PAINS	1 alert: catechol_A
Brenk	1 alert: catechol
Leadlikeness	No; 1 violation: XLOGP3>3.5
Synthetic accessibility	3.84

Figure S25. ADME-Tox and physicochemical properties of **13** predicted using the SwissADME web server (www.swissadme.ch).

Table S1. Predicted pharmacokinetics parameters of the examined compounds obtained by using the SwissADME server (www.swissadme.ch).

**Compound**	**GI absorption**	**BBB permeant**	**Pgp substrate**	**CYP1A2 inhibitor**	**CYP2C19 inhibitor**	**CYP2C9 inhibitor**	**CYP2D6 inhibitor**	**CYP3A4 inhibitor**	**log Kp (cm/s)**
**1**	High	Yes	No	No	Yes	Yes	Yes	No	-3,28
**2**	High	Yes	No	No	No	No	Yes	No	-4,55
**3**	High	Yes	Yes	Yes	Yes	Yes	Yes	Yes	-5,02
**4**	High	Yes	Yes	No	No	Yes	No	No	-5,21
**5**	High	Yes	Yes	No	Yes	Yes	No	Yes	-4,20
**6**	High	No	Yes	No	No	Yes	No	Yes	-4,24
**7**	High	No	Yes	No	No	Yes	No	Yes	-4,24
**8**	High	No	Yes	No	Yes	Yes	No	Yes	-4,40
**9**	High	No	Yes	No	Yes	Yes	No	Yes	-4,40
**10**	High	Yes	No	No	Yes	Yes	No	Yes	-5,45
**11**	High	Yes	No	No	Yes	Yes	No	No	-5,62
**12**	High	No	Yes	No	Yes	Yes	No	Yes	-4,55
**13**	High	Yes	No	No	No	Yes	Yes	No	-4,72
